# The influence of dietary intake of omega-3 polyunsaturated fatty acids on the association between short-term exposure to ambient nitrogen dioxide and respiratory and cardiovascular outcomes among healthy adults

**DOI:** 10.1186/s12940-021-00809-9

**Published:** 2021-12-07

**Authors:** Hao Chen, Siqi Zhang, Wan Shen, Claudia Salazar, Alexandra Schneider, Lauren Wyatt, Ana G. Rappold, David Diaz-Sanchez, Robert B. Devlin, James M. Samet, Haiyan Tong

**Affiliations:** 1 Oak Ridge Institute for Science and Education, 100 ORAU Way, 37830 Oak Ridge, TN USA; 2 Institute of Epidemiology, Helmholtz Zentrum München, Neuherberg, Germany; 3 Department of Public and Allied Health, Bowling Green State University, Bowling Green, OH USA; 4 Public Health and Integrated Toxicology Division, Center for Public Health and Environmental Assessment, Office of Research and Development, U.S. Environmental Protection Agency, 104 Mason Farm Rd, 27514 Chapel Hill, NC USA

**Keywords:** Air pollution, Nitrogen dioxide, Omega-3 polyunsaturated fatty acids, Lung function, Cardiovascular

## Abstract

**Background:**

Short-term exposure to ambient nitrogen dioxide (NO_2_) is associated with adverse respiratory and cardiovascular outcomes. Supplementation of omega-3 polyunsaturated fatty acids (PUFA) has shown protection against exposure to fine particulate matter. This study aims to investigate whether habitual omega-3 PUFA intake differentially modify the associations between respiratory and cardiovascular responses and short-term exposure to ambient NO_2_.

**Methods:**

Sixty-two healthy participants were enrolled into low or high omega-3 groups based on their habitual omega-3 PUFA intake. Each participant was repeatedly assessed for lung function, blood lipids, markers of coagulation and fibrinolysis, vascular function, and heart rate variability (HRV) in up to five sessions, each separated by at least 7 days. This study was carried out in the Research Triangle area of North Carolina, USA between October 2016 and September 2019. Daily ambient NO_2_ concentrations were obtained from an area air quality monitoring station on the day of outcome assessment (Lag0), 4 days prior (Lag1-4), as well as 5-day moving average (5dMA). The associations between short-term exposure to NO_2_ and the measured indices were evaluated using linear mixed-effects models stratified by omega-3 levels and adjusted by covariates including relative humidity and temperature.

**Results:**

The average concentration of ambient NO_2_ during the study periods was 5.3±3.8 ppb which was below the National Ambient Air Quality Standards (NAAQS). In the high omega-3 group, an interquartile range (IQR) increase in short-term NO_2_ concentrations was significantly associated with increased lung function [e.g. 1.2% (95%CI: 0.2%, 2.2%) in FVC at lag1, 2.6% (95%CI: 0.4%, 4.8%) in FEV1 at 5dMA], decreased blood lipids [e.g. -2.6% (95%CI: -4.4%, -0.9%) in total cholesterol at lag2, -3.1% (95%CI: -6.1%, 0.0%) in HDL at 5dMA, and -3.1% (95%CI: -5.5%, -0.7%) in LDL at lag2], improved vascular function [e.g. 8.9% (95%CI: 0.6%, 17.2%) increase in FMD and 43.1% (95%CI: -79.8%, -6.3%) decrease in endothelin-1 at 5dMA], and changed HRV parameters [e.g. -7.2% (95%CI: -13.6%, -0.8%) in HFn and 13.4% (95%CI: 0.2%, 28.3%) in LF/HF ratio at lag3]. In the low omega-3 group, an IQR increase in ambient NO_2_ was associated with elevations in coagulation markers (von Willebrand Factor, D-dimer) and a decrease in HRV (very-low frequency); however, null associations were observed between short-term NO_2_ exposure and changes in lung function, blood lipids, and vascular function.

**Conclusions:**

The results in this study imply that dietary omega-3 PUFA consumption may offer respiratory and vascular benefits in response to short-term exposure of healthy adults to NO_2_ levels below the NAAQS.

**Trial registration:**

ClinicalTrials.gov (NCT02921048).

**Supplementary Information:**

The online version contains supplementary material available at 10.1186/s12940-021-00809-9.

## Background

Respiratory diseases such as chronic obstructive pulmonary disease (COPD), lower respiratory infections, and lung cancers, and cardiovascular diseases such as ischemic heart disease and stroke, are worldwide leading causes of death [1]. Exposure to ambient air pollution has been associated with increased risk of respiratory and cardiovascular diseases and it is estimated to be responsible for 4.51 million deaths worldwide in 2019 [2].

Nitrogen dioxide (NO_2_) is a criteria air pollutant and formed from fossil fuel combustion in vehicular sources, power plants, and wildfires [3, 4]. Man-made sources in the U.S. are estimated to release approximately 12.3 million tons of nitrogen oxides (including NO_2_) in 2014 [5]. Short-term exposure to elevated concentrations of NO_2_ can cause airway irritation, exacerbate asthma and COPD, and increase hospital admissions due to respiratory or cardiovascular conditions [6, 7]. Although the implementation of the Clean Air Act has reduced ambient NO_2_ concentrations in the U.S., human exposure to NO_2_, especially at concentrations below the National Ambient Air Quality Standards (NAAQS) (1-hour: 100 ppb; annual: 53 ppb), is still associated with adverse health outcomes. For example, significant associations were observed between short-term or long-term exposure to ambient NO_2_ and all-cause cardiovascular and respiratory mortality [8–12]. Such adverse health impacts warrant further investigation of potential interventional approaches to mitigate the health effects of ambient NO_2_ exposure.

Dietary supplementation with fish oil from marine sources has been proposed as a potential intervention against the health effects of particulate matter [13]. Fish oil is rich in omega-3 polyunsaturated fatty acids (omega-3 PUFA), including eicosapentaenoic acid (EPA) and docosahexaenoic acid (DHA). EPA and DHA contain 5 and 6 carbon-carbon double bonds, respectively, and precursor for the synthesis of a class of lipid metabolites called specialized pro-resolving mediators (SPM) that orchestrate resolution of inflammation and a return to homeostasis [14, 15]. NO_2_ is a free radical species known to cause health effects through oxidative stress and inflammation [3]. Thus, the biochemical properties of omega-3 PUFA may offer protection against NO_2_ toxicity by direct reaction or by counteracting its pro-inflammatory effects. Previous studies have shown that omega-3 PUFA supplementation may lower the risk of cardiac dysrhythmia, inflammation, coagulopathy, endothelial dysfunction, and dyslipidemia induced by exposure to fine particulate matter (PM_2.5_) [16–18]. However, few have specifically investigated the respiratory and cardiovascular benefits of omega-3 PUFA against exposure to NO_2_ [11, 19]. To the best of our knowledge, this study is the first to investigate health benefits of omega-3 PUFA-rich diet against short-term exposure to ambient NO_2_ at concentrations below the NAAQS.

In the present study, healthy adult participants were enrolled based on their habitual omega-3 PUFA intake and stratified into low and high omega-3 PUFA groups. Focusing on lung function and subclinical cardiovascular parameters, this study aimed to investigate whether habitual dietary intake of omega-3 PUFA differentially modifies the associations between respiratory and cardiovascular responses and short-term exposure to ambient NO_2_.

## Methods

### Study participants and design

The study was conducted at the U.S. Environmental Protection Agency’s Human Studies Facility (HSF) in Chapel Hill, North Carolina, USA between October 2016 and September 2019, and all participants were residents of the Research Triangle area near HSF. Eligible participants meeting the following criteria were recruited: 25 – 55 years old; BMI between 19 and 35; having no history of respiratory or cardiovascular disease; non-smoker for at least one year; not taking β-adrenergic receptor blockers or anti-inflammatory drugs. Participants meeting at least one of the following criteria were enrolled into low or high omega-3 groups: (1) habitual dietary EPA + DHA intake ≤ 0.5 g/week (low) or ≥ 3.0 g/week (high) for at least six months based on a validated dietary questionnaire [20]; (2) red blood cell membrane omega-3 index ≤ 4.0% (low) or ≥ 5.5% (high) obtained from finger prick (OmegaQuant, Sioux Falls, SD). A total of 62 participants were enrolled into low (28) and high (34) omega-3 groups.

As shown in Additional Fig. 1, each participant had up to 5 study sessions separated by at least 7 days. Each session consisted of 2 consecutive days. On the first day, each participant was outfitted with a Holter monitor and recorded for 30 min while resting. On the second day, venous blood samples were collected, and spirometry, branchial artery ultrasound (BAU), 30-min Holter recordings were measured. All outcome measurements were conducted, and blood samples collected nearly same time of the day. Written informed consent was given by all participants prior to enrollment. The study was registered at ClinicalTrials.gov (NCT02921048) and approved by the Institutional Review Board of the University of North Carolina at Chapel Hill and the U.S. Environment Protection Agency.

### Exposure assessment

Hourly concentrations of ambient NO_2_ were obtained from the Millbrook air monitoring station close to the HSF. Twenty four-hour average concentrations of NO_2_ were calculated from the hourly pollutant data between 9 AM and 8 AM, with a valid day defined as having at least 18 hourly measurements over the 24-h period. Concentrations were assigned to each visit session (the day of blood sample collection as lag0), as well as to 4 days prior (lag1–lag4), and 5-day moving average (5dMA). Twenty four-hour averages of temperature and relative humidity were collected from the same monitoring station.

### Outcome assessment

#### Spirometry measurement

Spirometry was measured by a 10.2-L dry seal digital spirometer interfaced to a computer (SensorMedics Model 1022, SensorMedics, Palm Springs, CA). At least three sets of qualified data were obtained and the largest value was selected for forced vital capacity (FVC) and forced expiratory volume at the end of the first second (FEV1) as per American Thoracic Society guidelines [21].

#### Venous blood samples

A portion of whole blood samples was sent for lipid analysis (LabCorp, Burlington, NC). The other portion of blood samples was separated for plasma and stored at -80 ℃ prior to biomarker analysis. Commercially available multi-array plates were used to quantify levels of von Willebrand factor (vWF), tissue plasminogen activator (tPA), and D-dimer (MesoScale, Rockville, MD). Endothelin-1 was tested using an ELISA kit purchased from Peninsula Laboratories International (San Carlos, CA). All experiments were performed per manufacturers’ instructions.

#### Brachial artery ultrasound

Endothelial function was assessed by BAU using an Acuson Sequoia ultrasound machine (Siemens Healthcare, Malvern, PA) as described previously [17]. Briefly, resting blood pressure and baseline images of the right branchial artery at end diastole were captured. Flow–mediated dilation (FMD) was measured during reactive hyperemia induced by inflating a pneumatic tourniquet applied distal to the antecubital fossa to a suprasystolic pressure for 5 min. Hyperemic images were recorded for 90 s following cuff deflation. Brachial artery diameter (BAD) at baseline (BADb) and at maximum dilation (BADhyp) was measured using a customized software that utilizes edge-detection technology (Vascular Research Tools, Medical Imaging Applications, Coralville, IA).

#### Holter monitoring

HRV and repolarization parameters were measured using the last 5 min of Holter recording. Briefly, participants reclined in a dark room for 30 min and Holter were recorded using a H12+ 12-Lead ECG Recorder (Mortara, Milwaukee, WI). Assessed time-domain measurements include standard deviation of normal-to-normal (SDNN) and root-mean square of successive differences (rMSSD). Measured frequency-domain measurements include very-low frequency (VLF), normalized low frequency (LFn), normalized high frequency (HFn), and low-to-high frequency ratio (LF/HF).

### Covariates

Participant sociodemographic characteristics were obtained via a standardized interview by medical staff at the baseline visit. The collected information included age (years, continuous), sex (male or female), race/ethnicity (Caucasian, African-American, Asian, or others), marital status (single, married, separated, or divorced), and educational attainment (graduate degree, college degree, high school/trade school or lower). Besides, height (m, continuous) and weight (kg, continuous) were measured at baseline to calculate the body mass index (BMI) by BMI = weight / height^2^.

### Statistical analysis

A “gamm4” package in R (version 3.6.2) was employed to perform statistical analysis. Generalized linear mixed models with random participant effects were employed to analyze the association between short-term exposure to NO_2_ and the health parameters. Each visit of the participants was treated as a single data point. The dependent variables were log-transformed to improve normality in the residuals except for FEV1, FVC, FEV1/FVC, FMD, BADb, BADhyp, ET-1, LFn, and HFn. The statistical model was adjusted for age, sex, race, BMI, long-term and seasonal trends, day of the week, temperature, and relative humidity. The long-term and seasonal trends were controlled for by a penalized spline of time with eight degrees of freedom (df) per year. Temperature (lag0-1 for high temperatures and lag0-4 for low temperatures) and relative humidity (lag0-4) were incorporated as penalized splines with the df selected by the Generalized Cross Validation criterion. Linear terms of NO_2_ were included in the model separately to assess the immediate (lag0), delayed (lag1 to lag4), or cumulative (5dMA) effects. Between-group differences were assessed using a product term of omega-3 groups and air pollutant concentrations. The results were interpreted as percent change from the mean of the measured outcome per interquartile range (IQR) increase of NO_2_. Sensitivity analyses were conducted after excluding outcome outliers (defined as those lower than 1st Quartile-3×IQR and those higher than 3rd Quartile+3×IQR), using a 2-pollutant model with further adjustment for either PM_2.5_ or O_3_ concentrations obtained from the same air monitoring station, or adding additional covariates related with marital status and educational attainment. Statistical significance was set at a two-sided *p* < 0.05 for the air pollution effects and a two-sided p < 0.1 for the interaction with the two groups.

## Results

### Descriptive statistics

Twenty-eight participants in the low and 34 in the high omega-3 groups completed a total of 301 study sessions. Between the two groups, no statistical difference was observed in age, race / ethnicity, sex, smoking history, BMI, and systolic or diastolic blood pressure. The mean omega-3 index of the high omega-3 group was significantly higher than that of the low group (6.8% vs. 4.0%, *p* < 0.05) (Table 1). Descriptive statistics of all the outcome variables are summarized in Additional Table 1. During the study period, daily NO_2_ concentrations ranged from 0.8 to 24.2 ppb with a mean of 5.3 and an interquartile range (IQR) of 3.8 ppb. Temperature and relative humidity ranged from -8.6 to 31.1 °C, and 30 to 100%, respectively (Table 2). NO_2_ concentrations were weakly or moderately correlated with PM_2.5_, O_3_, or meteorological measurements. The correlations between PM_2.5_ and O_3_ concentrations and meteorological measurements were also considered weak or moderate (Additional Table 2).

**Table 1:** Participant characteristics

	Low omega-3 group (*n* = 28)	High omega-3 group (*n* = 34)	All (*n* = 62)
Age (years) mean (SD)	37 (8)	40 (9)	38 (9)
Sex n (%)			
Male	10 (35.7)	13 (38.2)	23 (37.1)
Female	18 (64.3)	21 (61.8)	39 (62.9)
Race / ethnicity n (%)			
African-American	9 (32.1)	5 (14.7)	14 (22.6)
Asian	0 (0)	3 (8.8)	3 (4.8)
Caucasian	19 (67.9)	26 (76.5)	45 (72.6)
Smoking history n (%)			
Nonsmoker	22 (78.6)	32 (94.1)	54 (87.1)
x-smoker	6 (21.4)	2 (5.9)	8 (12.9)
BMI (kg/m^2^) mean (SD)	24.9 (3.3)	24.4 (3.1)	24.6 (3.2)
Omega-3 index (%) mean (SD)	4.0 (0.8)	6.8 (1.2) ^*^	5.5 (1.7)
SBP (mmHg) mean (SD)	113.0 (8.8)	109.9 (9.9)	111.3 (9.5)
DBP (mmHg) mean (SD)	71.5 (6.7)	69.5 (7.3)	70.4 (7.1)

Statistical difference between low and high omega-3 groups was derived using Kruskal-Wallis rank sum tests for continuous variables and Fisher’s exact tests for categorical variables *: *p* < 0.05 for the difference between groups. BMI: body mass index, DBP: diastolic blood pressure, SBP: systolic blood pressure, SD: standard deviation

**Table 2:** Distribution of ambient NO_2_ concentrations and meteorological conditions from Oct. 6 2016 to Sep. 5 2019

	Mean (SD)	Range	IQR
NO_2_ (ppb)	5.3 (3.8)	0.8 – 24.2	3.8
Temperature (°C)	16.5 (8.9)	-8.6 – 31.1	15.2
Relative humidity (%)	70.2 (15.6)	30 – 100	22.2

IQR: interquartile range, SD: standard deviation

## Overview of findings

As summarized in Table 3, in the low omega-3 group, lung function and most cardiovascular markers were either not affected by an IQR increase in short-term NO_2_ concentrations or altered in an adverse direction. In contrast, in the high omega-3 group, significant associations were observed between short-term exposure to NO_2_ and increased lung function, decreased blood lipids, and increased vascular function. The magnitude and direction of the associations in the two groups varied by endpoints and exposure lags. We only report outcomes with significant percent change (with 95% CI) per an IQR increase of ambient NO_2_ concentration and significant interaction results (*p*_*interaction*_) in the following sections. All detailed results for each outcome variable are available in the Additional materials.

**Table 3:** Summary of statistical model results

Outcome	Low omega-3 group	
Lung function		
FVC	$\to$	$\uparrow_{L0-2\;and\;5dMA}$
FEV1	$\to$	${\uparrow }_{5dMA}$
Blood lipids		
Total cholesterol	$\to$	${\downarrow }_{L2}$
HDL	$\to$	$\downarrow_{L1-2\;and\;5dMA}$
LDL	$\to$	${\downarrow }_{L2}$
Coagulation / fibrinolysis factors		
vWF	${\uparrow }_{L0}$	$\uparrow_{L1\;and\;5dMA}$
D-dimer	${\uparrow }_{L0}$	$\to$
Vascular function		
FMD	$\to$	$\uparrow_{L1-2\;and\;5dMA}$
ET-1	${\downarrow }_{5dMA}$	$\downarrow_{L3-4\;and\;5dMA}$
Heart rate variability		
HFn	$\to$	${\downarrow }_{L3}$
LF/HF	$\to$	${\uparrow }_{L3}$
VLF	${\downarrow }_{L0}$	$\to$

Arrows “↓ ↑ and →” indicate negative, positive, or null associations between NO_2_ exposure and respiratory and cardiovascular parameters, respectively. 5dMA: 5-day moving average, ET-1: endothelin 1, FEV1: forced expiratory volume at the end of the first second, FMD: flow-mediated dilation, FVC: forced vital capacity, HDL: high density lipoprotein, HFn: normalized high frequency, L0: lag0, L1: lag1, L2: lag2, L3: lag3, L4: lag4, LDL: low density lipoprotein, LF/HF: low-to-high frequency ratio, VLF: very-low frequency, vWF: von Willebrand factor

### Lung function

**Fig. 1:**
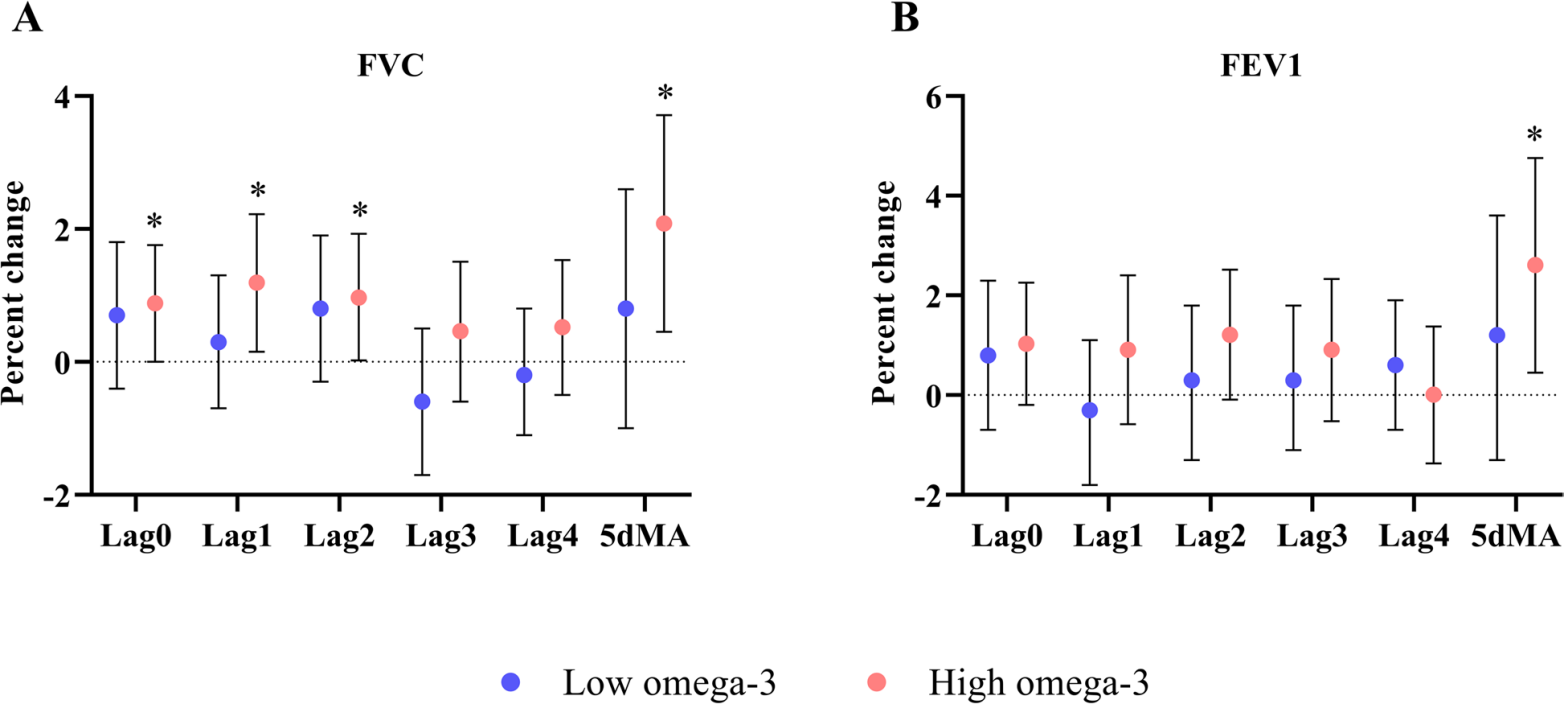
Effects of omega-3 PUFA on lung function in response to short-term exposure to ambient NO_2_. Effect estimates (95% CI) in FVC (**A**) and FEV1 (**B**) were calculated relative to an IQR increase in NO_2_ concentrations at lag 0 to 4 days, as well as 5-day moving average in the low and high omega-3 groups. * *p* < 0.05 for significant association within a group. FVC: force vital capacity, FEV1: forced expiratory volume at the end of the first second, IQR: interquartile range

No significant association between short-term exposure to NO_2_ concentrations and lung function was observed in the low omega-3 group. In contrast, in the high omega-3 group, an IQR increase in NO_2_ concentration was associated with statistically significant increases in FVC at lag0 [0.9% (0, 1.8%)], lag1 [1.2% (0.2%, 2.2%)], lag2 [1.0% (0.02%, 1.9%)], and 5dMA [2.1% (0.5%, 3.7%)] (Fig. 1 A), and with an increase in FEV1 at 5dMA [2.6% (0.5%, 4.8%)] (Fig. 1B). We did not detect significant associations in FEV1/FVC ratio; and no between-group differences were observed for any of the three lung function parameters (Table 3).

### Blood lipids

**Fig. 2:**
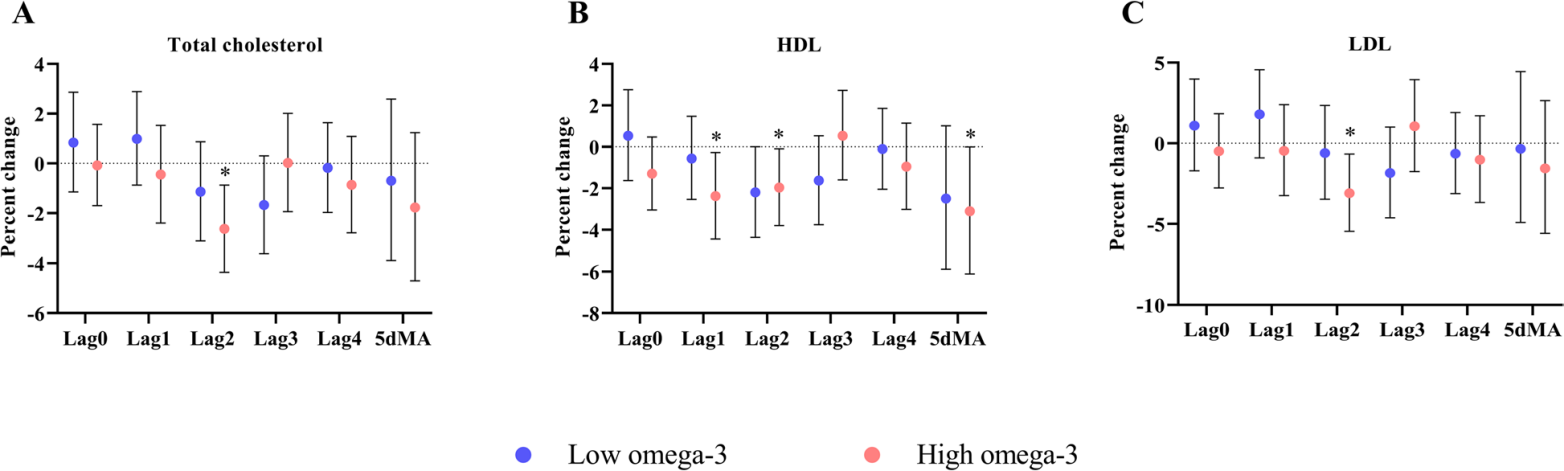
Effects of omega-3 PUFA on blood lipids in response to short-term exposure to ambient NO_2_. Effect estimates (95% CI) in total cholesterol (**A**), HDL (**B**), and LDL (**C**) were calculated relative to an IQR increase in NO_2_ concentrations at lag 0 to 4 days, as well as 5-day moving average in the low and high omega-3 groups. * *p* < 0.05 for significant association within a group. HDL: high-density lipoprotein, IQR: interquartile range, LDL: low-density lipoprotein

In the low omega-3 group, an IQR increase in NO_2_ concentrations was not associated with changes in blood lipids. However, in the high omega-3 group, NO_2_ exposure was significantly associated with reductions in total cholesterol at lag2 [-2.6% (-4.4%, -0.9%)], LDL at lag2 [-3.1% (-5.5%, -0.7%)], and HDL at lag1 [-2.4% (-4.4%, -0.3%)], lag2 [-2.0% (-3.8%, -0.1%)], and 5dMA [-3.1% (-6.1%, -0.01%)]. We did not observe significant between-group differences (Fig. 2, Additional Table 4).

### Coagulation and fibrinolysis

**Fig. 3:**
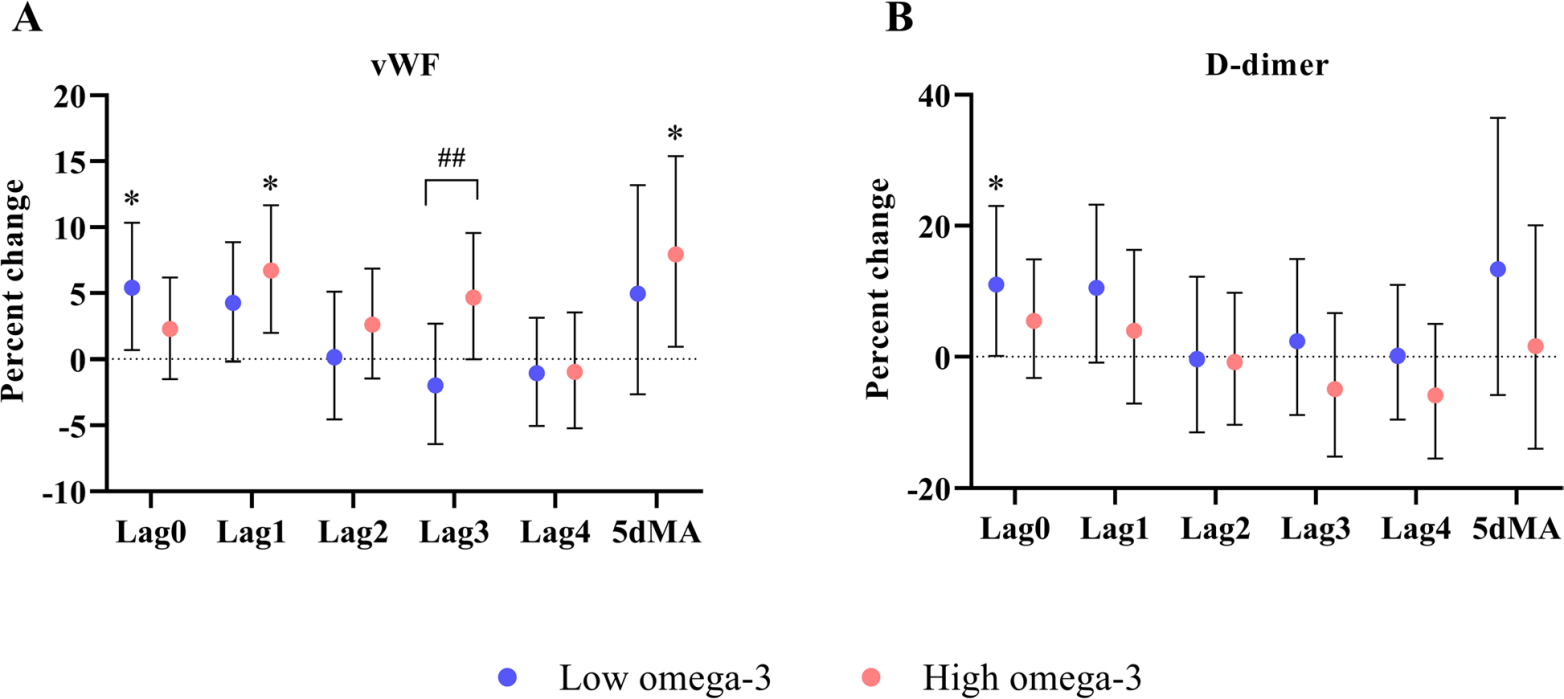
Effects of omega-3 PUFA on coagulation and fibrinolysis markers in response to short-term exposure to ambient NO_2_. Effect estimates (95% CI) in vWF (**A**) and D-dimer (**B**) were calculated relative to an IQR increase in NO_2_ concentrations at lag 0 to 4 days, as well as 5-day moving average in the low and high omega-3 groups. * *p* < 0.05 for significant association within a group. ^#^ *p*_interaction_ < 0.1 and ^##^ *p*_interaction_ < 0.05 for significant differences in the effect estimates between groups. IQR: interquartile range, vWF: von Willebrand factor

In the low omega-3 group, short-term NO_2_ exposure was associated with increases in D-dimer [11.0% (0.2%, 23.0%)] and vWF [5.4% (0.7%, 10.3%)] at lag0. In the high omega-3 group, NO_2_ exposure was associated with an increase in vWF levels at lag1 [6.7% (2.0%, 11.7%)], lag3 [4.7% (0.0, 9.6%), *p*_interaction_ = 0.026], and 5dMA [7.9% (1.0%, 15.4%)] (Fig. 3). No other significant associations or between-group differences were observed (Additional Table 5).

### Vascular function

**Fig. 4:**
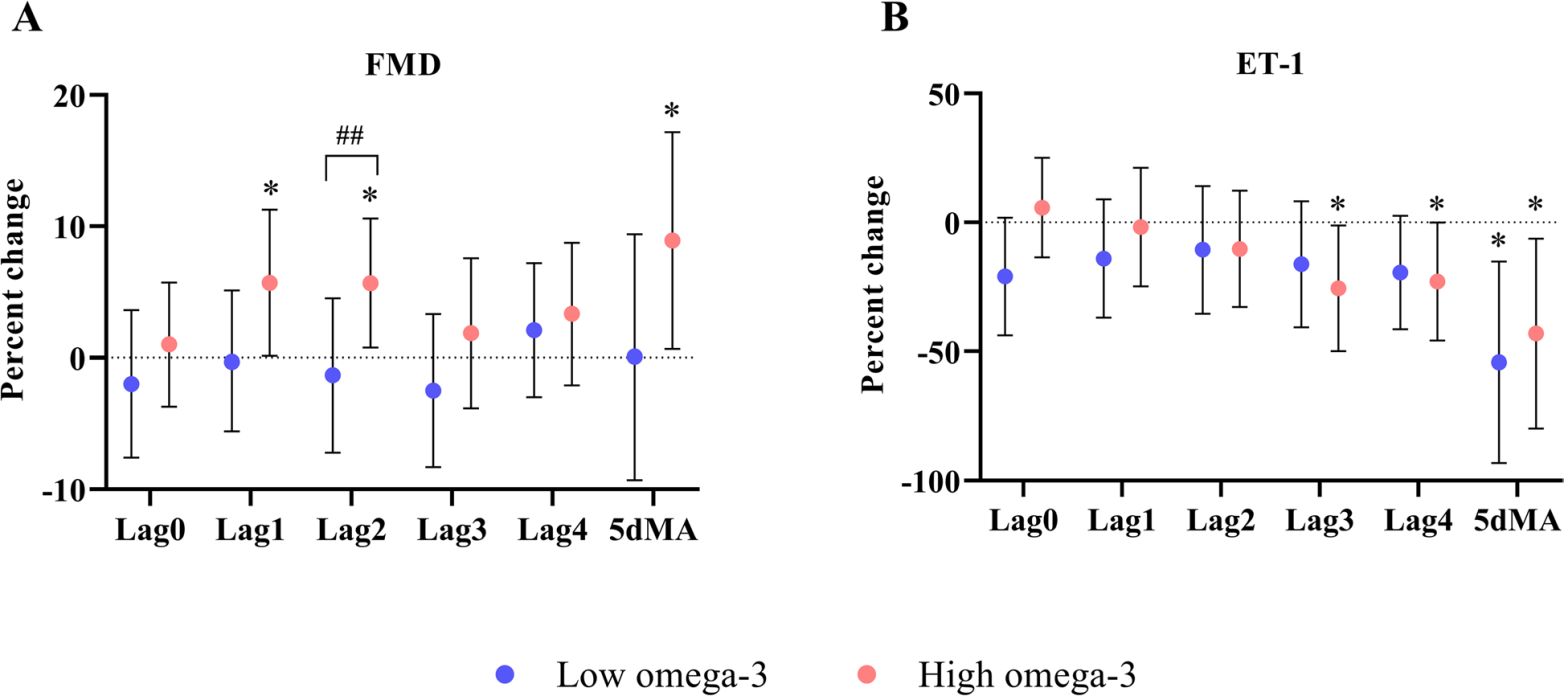
Effects of omega-3 PUFA on endothelial function markers in response to short-term exposure to ambient NO_2_. Effect estimates (95% CI) in FMD (**A**) and ED-1 (**B**) were calculated relative to an IQR increase in NO_2_ concentrations at lag 0 to 4 days, as well as 5-day moving average in the low and high omega-3 groups. * *p* < 0.05 for significant association within a group. ^#^ *p*_interaction_ < 0.1 and ^##^ *p*_interaction_ < 0.05 for significant differences in the effect estimates between groups. ED-1: endothelin 1, FMD: flow-mediated dilation, IQR: interquartile range

Short-term NO_2_ exposure was significantly associated with increases in FMD at lag1 [5.7% (0.1%, 11.2%)], lag2 [5.7% (0.8%, 10.6%), *p*_interaction_ = 0.044], and 5dMA [8.9% (0.6%, 17.2%)] in the high omega-3 group, while no significant association was observed in the low group. Significant associations were observed between NO_2_ exposure and decreased ET-1 levels at lag3 [-25.6% (-50.0%, -1.2%)], lag4 [-22.9% (-45.9%, -0.01%)], and 5dMA [-43.1% (-79.8%, -6.3%)] in the high omega-3 group as well as at 5dMA [-54.2% (-93.2%, -15.2%)] in the low group (Fig. 4). We did not observe any other NO_2_ – associated changes in either group (Additional Table 6).

### Heart rate variability

**Fig. 5:**
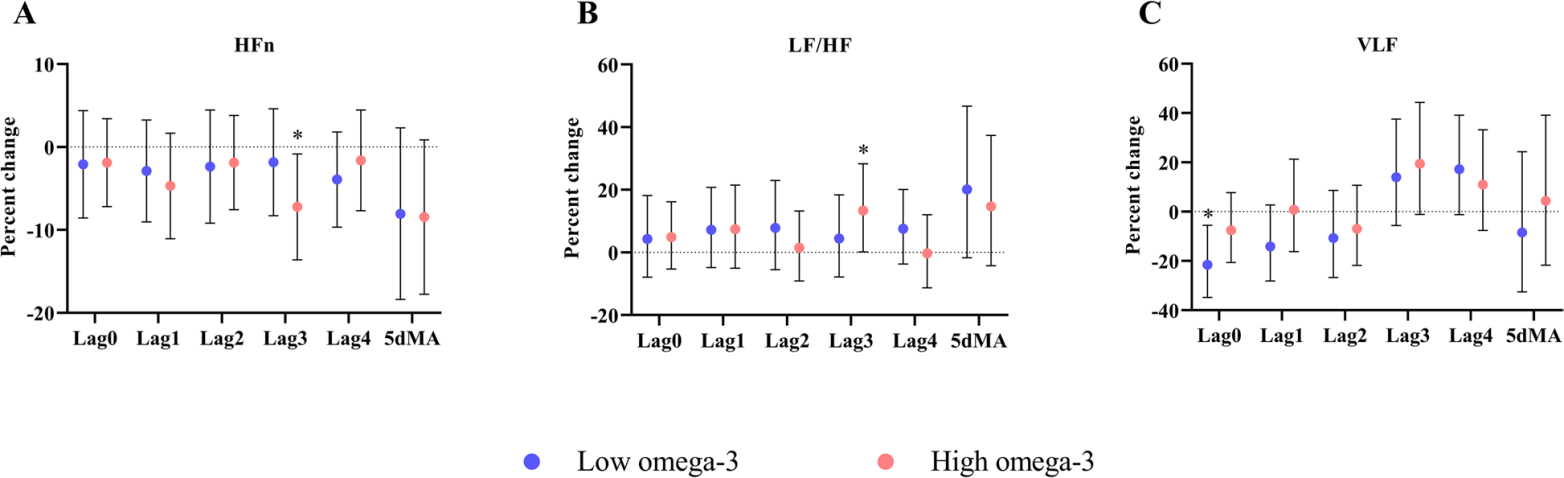
Effects of omega-3 PUFA on HRV markers in response to short-term exposure to ambient NO_2_. Effect estimates (95% CI) in HFn (**A**), LF/HF (**B**), and VLF (**C**) were calculated relative to an IQR increase in NO_2_ concentrations at lag 0 to 4 days, as well as 5-day moving average in the low and high omega-3 groups.  *p* < 0.05 for significant association within a group. HFn: normalized high frequency, IQR: interquartile range, LF/HF: low-to-high frequency ratio, VLF: very low frequency

In the low omega-3 group, short-term NO_2_ exposure was significantly associated with decreased VLF at lag0 [-21.5% (-34.8%, -5.6%)]. In contrast NO_2_ exposure was associated with decreased HFn [-7.2% (-13.6%, - 0.8%)] and increased LF/HF ratio [13.4% (0.2%, 28.3%)] at lag3 in the high omega-3 group (Fig. 5). No other significant associations or between-group differences were observed in HRV parameters (Additional Table 7).

### Sensitivity analysis

The associations between short-term exposure to NO_2_ and the biomarkers were robust in the two-air pollutant model after adjustment for simultaneous exposure to PM_2_._5_ or ozone (Additional Table 8). In addition, the results remained stable after excluding outliers of the endpoints indicating that the statistically significant associations were not driven by them (Additional Table 9). Finally, we included marital status and education level as covariates in the statistical model and the overall results did not change, indicating that these socioeconomic proxies did not confound the overall findings (Additional Table 10).

## Discussion

In this panel study, we investigated the modulative effects of omega-3 PUFA on the association between respiratory and cardiovascular effects and exposure to ambient NO_2_ in healthy participants. We observed that short-term exposure to ambient NO_2_ was associated with increased lung function and endothelial function, and lowered blood lipids among participants with high omega-3 PUFA levels.

NO_2_ is a gaseous and oxidant pollutant and inhalational exposure to ambient NO_2_ can induce oxidative stress and pulmonary inflammation [19, 22]. Omega-3 PUFA from marine sources may block oxidative damage to cells by acting as a target for reaction with NO_2_ [14], and increased availability of EPA and DHA may lead to higher levels of SPMs to promote resolution of inflammation caused by NO_2_ exposure. One randomized trial reported that fish oil supplementation reduced oxidative stress caused by short-term exposure to environmental oxidants including ozone and NO_2_ among young healthy participants [19]. Another cohort study found a beneficial effect of Mediterranean diet, rich in marine fish, against cardiovascular risk related to long-term exposure to NO_2_, although no clear role for omega-3 PUFA [11]. To our knowledge, the present study is the first to specifically investigate the modulating effects of dietary omega-3 PUFA on the association between respiratory and cardiovascular parameters and NO_2_ exposure in healthy adults.

In this study, exposure to low-level ambient NO_2_ was not associated with lung function changes among participants with low omega-3 PUFA. Although epidemiological evidence shows a possible link between ambient NO_2_ exposure and adverse respiratory effects [6, 23–25], null associations were observed between NO_2_ exposure and lung function reductions in human exposure studies [22]. For example, chamber studies reported that short-term exposure to 0.5-2.0 ppm NO_2_ did not induce any significant changes in lung function among healthy subjects [26, 27]. It is worth reiterating that the ambient NO_2_ concentration in the present study is much lower than the tested chamber levels as well as the current NAAQS and is therefore not expected to induce a significant respiratory response.

Paradoxically, NO_2_ exposure in this study was associated with significant increases in FVC and FEV1 among participants with high omega-3 PUFA levels. One possible explanation may be found in the reactivity of NO_2_ with the electron-rich centers in unsaturated fatty acids, such as EPA and DHA [28]. Rich in oxidizable carbon-carbon double bonds, EPA and DHA can react with NO_2_ to generate nitroalkene derivative, nitro EPA (NO_2_-EPA) and nitro DHA (NO_2_-DHA) [28]. These nitroalkenes can further decay or possibly be metabolized via a reaction facilitated by reductants such as ascorbate to release the gasotransmitter nitric oxide (NO), a potent receptor-mediated stimulus that acts through a specific signaling pathway to promote the relaxation of smooth muscle cells [29–31]. It is therefore tempting to speculate that the improvements in vascular and lung function associated with NO_2_ exposure are underlain by vasodilatory and bronchodilatory changes in smooth muscle tone, respectively, that are mediated by an increased availability of NO in the tissues of the participants with high omega-3 in this study.

In addition to increased FMD, decreased plasma ET-1 were observed in the high omega-3 group in association with NO_2_ exposure. While FMD reflects dilation of the brachial artery caused by NO released from endothelial cells in response to shear-stress, ET-1 is a potent endogenous vasoconstrictor that is inhibited by NO [32–34]. Short-term exposure to NO_2_ is not known to be associated with endothelial dysfunction or vascular constriction [35]; however, as suggested earlier, NO_2_-EPA and NO_2_-DHA could be formed when NO_2_ reacts with omega-3 PUFA creating a releasable source of NO that may have resulted in the observed improvement in endothelial function in the high omega-3 group [28–30]. Studies to validate the apparent beneficial effects of NO_2_ in subjects with high omega-3 and to investigate the mechanistic basis are warranted.

Significant changes in blood lipids were also only observed in the high omega-3 group, indicating that high omega-3 PUFA intake may lower blood cholesterol levels in response to NO_2_ exposure. In a manner analogous to the reaction with fatty acids, the reaction of NO_2_ on cholesterol can lead to formation of cholesteryl nitrite [36]. It is possible that the presence of omega-3 PUFA may promote the nitration reaction leading to decreased levels of both the “good” (HDL) and the “bad” (total cholesterol and LDL) cholesterols. Among the blood coagulation markers studied, vWF binds to factor VIII and promotes platelet adhesion to injured vasculature while D-dimer is a product of the fibrin degradation process [37]. The NO_2_-associated elevation in vWF and D-dimer levels at lag0 in the low omega-3 group indicate acute coagulation and fibrinolysis in response to NO_2_ exposure. However, the lag1 and cumulative (5dMA) impacts of ambient NO_2_ on vWF found in the high omega-3 group suggest that the oxidation of omega-3 PUFA by NO_2_ could be a double-edged sword as it could counteract the acute toxicity of NO_2_, but also lead to activation of pro-thrombotic pathways.

HFn is an index of heart rate variability that reflects parasympathetic activity specifically correlating heart rate variations related to the respiratory cycle [38]. Similarly, the LF/HF ratio measures the “sympatho-vagal balance” with a high LF/HF ratio indicating sympathetic dominance [38]. Low VLF power is associated with several adverse health outcomes including arrhythmic death and high levels of inflammation [38]. A cohort study showed that each 10 µg/m^3^ (approximately 5 ppb) increment in the average yearly NO_2_ concentration was associated with decreases in SDNN, LFn, and LF/HF ratio in elderly women [39]. In a controlled exposure study, NO_2_ at 500 ppb increased HFn 1-hour post-exposure in young healthy participants [26]. In the present study, we observed that in the low omega-3 group, NO_2_ was associated with a decrease in VLF; while in the high group, NO_2_ was associated with a significant reduction in HFn and elevation in LF/HF ratio. These results suggest that high omega-3 PUFA may modulate baroreflex and parasympathetic activities in response to NO_2_ exposure.

To our knowledge, this study is the first to suggest health benefits of omega-3 PUFA-rich diet against short-term exposure to low-level ambient NO_2_. In addition, in this study, we evaluated respiratory and cardiovascular effects of NO_2_ exposure using multiple lags of exposure as well as a cumulative effect of NO_2_ using a 5-day moving average. Although we only measured omega-3 index at the enrollment phase and did not monitor omega-3 index throughout the study period, dietary intake of EPA+DHA for each participant was recorded at each study session using a 24-hour dietary recall methodology, and the results indicate that the omega-3 PUFA intake levels maintained for both low and high omega-3 groups throughout the study (Lukens MK, Kerri L, Tong H, Hao C, Shen W: A comparison of Omega-3 Fatty Acids Intakes from Three Dietary Screening Tools, unpublished).

This study also has some limitations. First, although this observational study was a longitudinal design, it employed a relatively small sample size, thus caution is advised establishing causal inference of the findings. Second, this study used central air monitors rather than personal monitors for air pollution data, which could possibly introduce non-differential exposure misclassification and bias the effects towards the null. Third, we only recruited healthy participants, and it is likely that additional and larger effects could be observed among more susceptible subgroups. Fourth, it is also possible that volatile organic compounds could be confounding factors since they are highly correlated with NO_2_ production. In addition, some of the significant findings could be by chance as the significant association was only reported at 1 lag day.

## Conclusions

This panel study suggests that participants with low intake of dietary omega-3 PUFA showed minimal respiratory effects in response to short-term exposure to ambient NO_2_ at concentrations below the NAAQS. In contrast, associations between improved pulmonary and vascular function and reduced blood lipid levels, and NO_2_ exposure were observed among participants with relatively high omega-3 PUFA. These findings suggest that increased dietary intake of omega-3 PUFA may offer health benefits against the impacts of short-term NO_2_ exposure in healthy adults.

## Supplementary Information

**Additional file 1.** Additional Figure 1 and Table 1-10.

## Data Availability

The datasets used and/or analysed during the current study are available from the corresponding author on reasonable request.

## Abbreviations

BAU: branchial artery ultrasound
CI: confidence interval
DHA: docosahexaenoic acid
ET-1: endothelin-1
EPA: eicosapentaenoic acid
FEV1: forced expiratory volume at the end of the first second
FMD: flow – mediated dilation
FVC: forced vital capacity
HDL: high-density lipoproteins
HFn: normalized high frequency
HRV: heart rate variability
LDL: low-density lipoproteins
LFn: normalized low frequency
LF/HF: low to high frequency ratio
NAAQS: National Ambient Air Quality Standards
NO_2_: nitrogen dioxide
PUFA: polyunsaturated fatty acids
RMSSD: root mean square of successive differences
SD: standard deviation
SDNN: standard deviation of normal-to-normal beats interval
tPA: tissue plasminogen activator
VLF: very-low frequency
vWF: von Willebrand factor
