# Supplementary material for: The influence of dietary intake of omega-3 polyunsaturated fatty acids on the association between short-term exposure to ambient nitrogen dioxide and respiratory and cardiovascular outcomes among healthy adults
Source: Environ Health. 2021 Dec 7;20:123. doi: 10.1186/s12940-021-00809-9 (PMC8650256; doi:10.1186/s12940-021-00809-9)
Supplement: Supplementary file 1 — Additional file 1. Additional Figure 1 and Table 1-10. [file 12940_2021_809_MOESM1_ESM.docx]

**Additional materials**

**The influence of dietary intake of omega-3 polyunsaturated fatty acids on the association between short-term exposure to ambient nitrogen dioxide and respiratory and cardiovascular outcomes among healthy adults**

Hao Chen, PhD ^a^; Siqi Zhang, PhD ^b^; Wan Shen, PhD ^a,c^; Claudia Salazar, BS ^d^, Alexandra Schneider, PhD ^b^; Lauren Wyatt, PhD ^d^; Ana G. Rappold, PhD ^d^; David Diaz-Sanchez, PhD ^d^; Robert B. Devlin, PhD ^d^; James M. Samet, MPH, PhD ^d^; Haiyan Tong, MD, PhD ^d^

^a^ Oak Ridge Institute for Science and Education, Oak Ridge, TN, USA. ^b^ Institute of Epidemiology, Helmholtz Zentrum München, Neuherberg, Germany. ^c^ Department of Public and Allied Health, Bowling Green State University, Bowling Green, OH, USA. ^d^ Public Health and Integrated Toxicology Division, Center for Public Health and Environmental Assessment, Office of Research and Development, U.S. Environmental Protection Agency, Chapel Hill, NC, USA.

**Additional Figure 1**. Schematic showing the study design. Eligible participants were 22 – 55 years old and generally healthy. Participants meeting at least one of the following criteria were enrolled into low or high omega-3 groups: 1) habitual dietary eicosapentaenoic acid (EPA) + docosahexaenoic acid (DHA) intake ≤ 0.5 g/week (low) or ≥ 3.0 g/week (high) for at least six months based on a validated dietary questionnaire; 2) omega-3 index ≤ 4.0% (low) or ≥ 5.5% (high). A total of 62 participants were enrolled into low (28) and high (34) omega-3 groups. Each participant had up to 5 study sessions separated by at least one week. Each session consisted of 2 consecutive days. On the first day, each participant completed 24-hour dietary recall and was outfitted with a Holter monitor and recorded for 30 min while resting. The first day visit on the first session was longer due to completing additional dietary questionnaire and spirometry training. On the second day visit, venous blood samples were collected, and spirometry, branchial artery ultrasound (BAU), 30-min Holter recordings were measured. The study was conducted in the Human Studies Facility of US EPA in Chapel Hill, North Carolina, USA between October 2016 and September 2019.

**
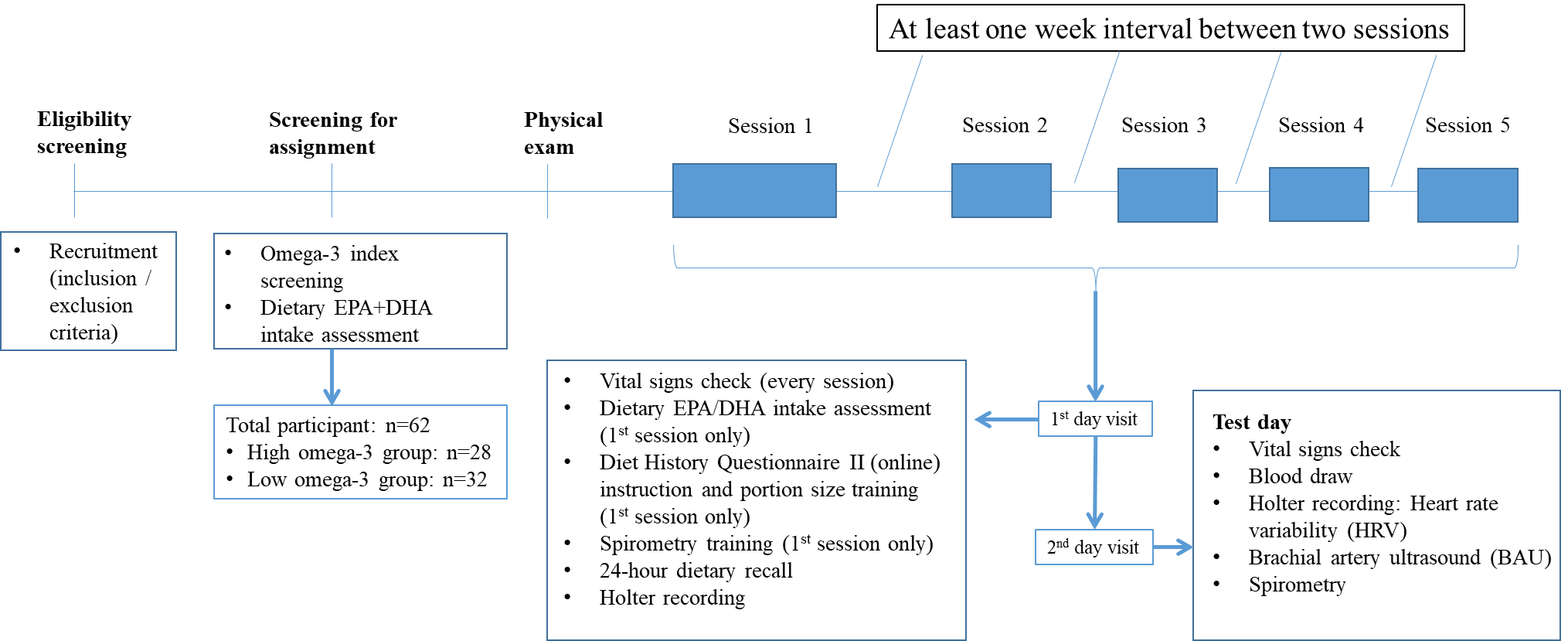
**

**Additional Table 1**. Descriptive statistics of all outcome variables [Mean (SD)].

| Outcome | Low omega-3 group  (*n* = 28) | | High omega-3 group  (*n* = 34) | All  (*n* = 62) | | |  |  |  |
| --- | --- | --- | --- | --- | --- | --- | --- | --- | --- |
| Lung function | | | | | | |  |  |  |
| FEV1 (L) | 3.3 (0.9) | | 3.3 (0.7) | 3.3 (0.8) | |  |  |  |  |
| FVC (L) | 4.2 (1.1) | | 4.2 (0.9) | 4.2 (1) | |  |  |  |  |
| FEV1/FVC (%) | 77.7 (6.5) | | 79.2 (6.2) | 78.5 (6.4) | |  |  |  |  |
| Blood lipids | | | | | | |  |  |  |
| Cholesterol (mg/dL) | 185.2 (36) | 190 (36.2) | | | 187.8 (36.1) | | |  |  |
| HDL (mg/dL) | 57.8 (15.1) | 63.6 (16.9) | | | 61 (16.4) | | |  |  |
| LDL (mg/dL) | 106.4 (33.6) | 107 (28.1) | | | 106.7 (30.7) | | |  |  |
| Coagulation / fibrinolysis | | | | | | |  |  |  |
| tPA (ng/mL) | 7.3 (5.1) | 7.4 (5.2) | | | 7.4 (5.1) | | |  |  |
| vWF (ng/mL) | 101 (44.6) | 107.4 (41.2) | | | 104.5 (42.8) | | |  |  |
| D-dimer (ng/mL) | 2365.2 (9531.6) | 1698.9 (2366.4) | | | 2002.2 (6658.7) | | |  |  |
| Endothelial function | | | | | | | | | |
| FMD (%) | 6.1 (2.9) | 8 (2.7) | | | 7.1 (2.9) | | |  |  |
| BADb (mm) | 3.4 (0.5) | 3.5 (0.5) | | | 3.4 (0.5) | | |  |  |
| BADhyp (mm) | 3.6 (0.5) | 3.8 (0.5) | | | 3.7 (0.5) | | |  |  |
| ET-1 (ng/ml) | 0.3 (0.27) | 0.3 (0.31) | | | 0.3 (0.29) | | |  |  |
| Heart rate variability | | | | | | | | |  |
| SDNN (ms) | 66.6 (21.3) | 72 (32.5) | | | 69.6 (28.1) | | |  |  |
| RMSSD (ms) | 44 (24.2) | 50.4 (35.1) | | | 47.5 (30.8) | | |  |  |
| HFn | 38.4 (16.6) | 37.7 (15.7) | | | 38 (16.1) | | |  |  |
| LFn | 50.6 (16.6) | 52 (15.3) | | | 51.4 (15.9) | | |  |  |
| LF/HF | 1.9 (1.7) | 1.9 (1.6) | | | 1.9 (1.7) | | |  |  |
| VLF (ms**2) | 1774.6 (1799.4) | 2359.6 (2904.9) | | | 2095 (2480.3) | | |  |  |

Note: There are a total of 301 study sessions. Among them, 3 subjects completed 3 sessions, 3 subjects completed 4 sessions, and 56 subjects completed 5 sessions. BADb: brachial artery diameter at baseline, BADhyp: brachial artery diameter at maximum dilatation, ET-1: endothelin-1, FEV1: forced expiratory volume at the end of the first second, FMD: Flow – mediated dilatation, FVC: forced vital capacity, HDL: high-density lipoproteins, HFn: normalized high frequency, LDL: low-density lipoproteins, LFn: normalized low frequency, LF/HF: low to high frequency ratio, RMSSD: root mean square of successive differences, SD: standard deviation, SDNN: standard deviation standard deviation of normal-to-normal, tPA: tissue plasminogen activator, VLF: very-low frequency, vWF: von Willebrand factor.

**Additional Table 2**. Distribution and Spearman correlation coefficients of exposure variables during the study period (Oct. 6, 2016 - Sep. 5, 2019)

|  | Mean (SD) | Range | IQR | Spearman correlation coefficient | | | |
| --- | --- | --- | --- | --- | --- | --- | --- |
|  |  |  |  | PM_2.5_ | O_3_ | NO_2_ | Temperature |
| PM_2.5_ (µg/m^3^) | 10.2 (4.1) | 1.8 – 68.0 | 4.7 |  |  |  |  |
| O_3_ 8h (ppb) | 40.8 (11.1) | 10 – 71 | 17 | 0.16 |  |  |  |
| NO_2_ (ppb) | 5.3 (3.8) | 0.8 – 24.2 | 3.8 | 0.45 | -0.13 |  |  |
| Temperature (°C) | 16.5 (8.9) | -8.6 – 31.1 | 15.2 | -0.10 | 0.47 | -0.42 |  |
| Relative humidity (%) | 70.2 (15.6) | 30 – 100 | 22.2 | -0.19 | -0.46 | -0.21 | 0.17 |
| Note: IQR: interquartile range, PM: particulate matter, SD: standard deviation. | | | | | | | |

**Additional Table 3**. Percent change (95% CI) in lung function parameters per IQR increase in NO_2_ in the low and high omega-3 groups.

| Outcome | Lag (Day) | Low omega-3 group | High omega-3 group | *p*_interaction_ |
| --- | --- | --- | --- | --- |
| FEV1 | Lag0 | 0.8 (-0.7, 2.3) | 1.0 (-0.2, 2.3) | 0.821 |
|  | Lag1 | -0.3 (-1.8, 1.1) | 0.9 (-0.6, 2.4) | 0.193 |
|  | Lag2 | 0.3 (-1.3, 1.8) | 1.2 (-0.1, 2.5) | 0.302 |
|  | Lag3 | 0.3 (-1.1, 1.8) | 0.9 (-0.5, 2.3) | 0.522 |
|  | Lag4 | 0.6 (-0.7, 1.9) | 0.0 (-1.4, 1.4) | 0.497 |
|  | 5dMA | 1.2 (-1.2, 3.6) | 2.6 (0.4, 4.8) | 0.292 |
| FVC | Lag0 | 0.7 (-0.4, 1.8) | 0.9 (0.0, 1.8) | 0.790 |
|  | Lag1 | 0.3 (-0.7, 1.3) | 1.2 (0.2, 2.2) | 0.181 |
|  | Lag2 | 0.8 (-0.3, 1.9) | 1.0 (0.0, 1.9) | 0.827 |
|  | Lag3 | -0.6 (-1.7, 0.5) | 0.5 (-0.6, 1.5) | 0.116 |
|  | Lag4 | -0.2 (-1.1, 0.8) | 0.5 (-0.5, 1.5) | 0.280 |
|  | 5dMA | 0.8 (-1.0, 2.6) | 2.1 (0.4, 3.7) | 0.207 |
| FEV1/FVC | Lag0 | -0.1 (-0.8, 0.5) | -0.1 (-0.6, 0.4) | 0.962 |
|  | Lag1 | -0.5 (-1.1, 0.1) | -0.1 (-0.7, 0.5) | 0.276 |
|  | Lag2 | -0.5 (-1.1, 0.2) | 0.0 (-0.5, 0.6) | 0.171 |
|  | Lag3 | 0.3 (-0.4, 0.9) | 0.1 (-0.6, 0.7) | 0.596 |
|  | Lag4 | 0.1 (-0.4, 0.7) | 0.1 (-0.5, 0.7) | 0.951 |
|  | 5dMA | -0.3 (-1.3, 0.7) | 0.1 (-0.8, 1.0) | 0.492 |

Note: 5dMA: 5-day moving average, CI: confidence interval, FEV1: forced expiratory volume at the end of the first second, FVC: forced vital capacity, IQR: interquartile range.

**Additional Table 4**. Percent change (95% CI) in blood lipid markers per IQR increase in NO_2_ in the low and high omega-3 groups.

| Outcome | Lag (Day) | Low omega-3 group | High omega-3 group | *p*_interaction_ |
| --- | --- | --- | --- | --- |
| Total cholesterol | Lag0 | 0.8 (-1.1, 2.9) | -0.1 (-1.7, 1.6) | 0.464 |
|  | Lag1 | 1.0 (-0.9, 2.9) | -0.5 (-2.4, 1.5) | 0.255 |
|  | Lag2 | -1.1 (-3.1, 0.9) | -2.6 (-4.4, -0.9) | 0.224 |
|  | Lag3 | -1.7 (-3.6, 0.3) | 0.0 (-1.9, 2.0) | 0.175 |
|  | Lag4 | -0.2 (-2.0, 1.6) | -0.9 (-2.8, 1.1) | 0.560 |
|  | 5dMA | -0.7 (-3.9, 2.6) | -1.8 (-4.7, 1.2) | 0.553 |
| HDL | Lag0 | 0.5 (-1.6, 2.8) | -1.3 (-3.1, 0.5) | 0.176 |
|  | Lag1 | -0.6 (-2.5, 1.5) | -2.4 (-4.4, -0.3) | 0.175 |
|  | Lag2 | -2.2 (-4.4, 0.0) | -2.0 (-3.8, -0.1) | 0.855 |
|  | Lag3 | -1.6 (-3.8, 0.5) | 0.5 (-1.6, 2.7) | 0.114 |
|  | Lag4 | -0.1 (-2.0, 1.9) | -1.0 (-3.0, 1.1) | 0.517 |
|  | 5dMA | -2.5 (-5.9, 1.0) | -3.1 (-6.1, 0.0) | 0.751 |
| LDL | Lag0 | 1.1 (-1.7, 4.0) | -0.5 (-2.8, 1.8) | 0.368 |
|  | Lag1 | 1.8 (-0.9, 4.6) | -0.5 (-3.2, 2.4) | 0.217 |
|  | Lag2 | -0.6 (-3.5, 2.3) | -3.1 (-5.5, -0.7) | 0.148 |
|  | Lag3 | -1.9 (-4.6, 1.0) | 1.1 (-1.8, 3.9) | 0.107 |
|  | Lag4 | -0.6 (-3.1, 1.9) | -1.0 (-3.7, 1.7) | 0.822 |
|  | 5dMA | -0.3 (-4.9, 4.4) | -1.6 (-5.6, 2.6) | 0.640 |

Note: 5dMA: 5-day moving average, CI: confidence interval, HDL: high-density lipoproteins, IQR: interquartile range, LDL: low-density lipoproteins.

**Additional Table 5**. Percent change (95% CI) in coagulation and fibrinolysis markers per IQR increase in NO_2_ in the low and high omega-3 groups.

| Outcome | Lag (Day) | Low omega-3 group | High omega-3 group | *p*_interaction_ |
| --- | --- | --- | --- | --- |
| tPA | Lag0 | 2.2 (-3.0, 7.5) | 2.5 (-1.7, 6.9) | 0.917 |
|  | Lag1 | 0.4 (-4.4, 5.4) | -0.4 (-5.3, 4.8) | 0.817 |
|  | Lag2 | 1.8 (-3.5, 7.3) | -1.7 (-5.9, 2.8) | 0.277 |
|  | Lag3 | 4.6 (-0.6, 10.0) | -2.9 (-7.7, 2.1) | 0.022 |
|  | Lag4 | 1.3 (-3.2, 6.1) | -0.5 (-5.3, 4.4) | 0.542 |
|  | 5dMA | 5.8 (-2.6, 15.0) | -0.1 (-7.2, 7.5) | 0.209 |
| vWF | Lag0 | 5.4 (0.7, 10.3) | 2.3 (-1.5, 6.2) | 0.295 |
|  | Lag1 | 4.3 (-0.1, 8.9) | 6.7 (2.0, 11.7) | 0.421 |
|  | Lag2 | 0.2 (-4.5, 5.1) | 2.6 (-1.5, 6.9) | 0.401 |
|  | Lag3 | -2.0 (-6.4, 2.7) | 4.7 (0.0, 9.6) | 0.026 |
|  | Lag4 | -1.0 (-5.0, 3.1) | -0.9 (-5.2, 3.5) | 0.973 |
|  | 5dMA | 5.0 (-2.6, 13.2) | 7.9 (0.9, 15.4) | 0.506 |
| D-dimer | Lag0 | 11.0 (0.2, 23.0) | 5.5 (-3.2, 14.9) | 0.421 |
|  | Lag1 | 10.5 (-0.8, 23.2) | 4.0 (-7.1, 16.3) | 0.395 |
|  | Lag2 | -0.3 (-11.5, 12.2) | -0.8 (-10.3, 9.8) | 0.946 |
|  | Lag3 | 2.4 (-8.8, 14.9) | -4.9 (-15.2, 6.7) | 0.313 |
|  | Lag4 | 0.2 (-9.5, 10.9) | -5.8 (-15.5, 5.0) | 0.366 |
|  | 5dMA | 13.4 (-5.8, 36.4) | 1.6 (-14.0, 20.1) | 0.279 |

Note: 5dMA: 5-day moving average, CI: confidence interval, IQR: interquartile range, tPA: tissue plasminogen activator, vWF: von Willebrand factor.

**Additional Table 6**. Percent change (95% CI) in endothelial function markers per IQR increase in NO_2_ in the low and high omega-3 groups.

| Outcome | Lag (Day) | Low omega-3 group | High omega-3 group | *p*_interaction_ |
| --- | --- | --- | --- | --- |
| FMD | Lag0 | -2.0 (-7.6, 3.6) | 1.0 (-3.7, 5.7) | 0.390 |
|  | Lag1 | -0.3 (-5.6, 5.1) | 5.7 (0.1, 11.2) | 0.095 |
|  | Lag2 | -1.3 (-7.2, 4.5) | 5.7 (0.8, 10.6) | 0.044 |
|  | Lag3 | -2.5 (-8.3, 3.3) | 1.9 (-3.8, 7.6) | 0.229 |
|  | Lag4 | 2.1 (-3.0, 7.2) | 3.3 (-2.1, 8.7) | 0.730 |
|  | 5dMA | 0.1 (-9.2, 9.4) | 8.9 (0.6, 17.2) | 0.083 |
| BADb | Lag0 | -0.2 (-1.0, 0.7) | -0.2 (-0.9, 0.4) | 0.882 |
|  | Lag1 | -0.5 (-1.3, 0.3) | 0.2 (-0.7, 1.0) | 0.236 |
|  | Lag2 | 0.0 (-0.9, 0.9) | -0.3 (-1.1, 0.4) | 0.528 |
|  | Lag3 | 0.4 (-0.5, 1.2) | -0.3 (-1.1, 0.5) | 0.202 |
|  | Lag4 | 0.6 (-0.2, 1.3) | -0.8 (-1.6, 0.0) | 0.008 |
|  | 5dMA | 0.0 (-1.4, 1.4) | -0.7 (-1.9, 0.5) | 0.344 |
| BADhyp | Lag0 | -0.3 (-1.1, 0.5) | -0.3 (-0.9, 0.4) | 0.967 |
|  | Lag1 | -0.5 (-1.3, 0.3) | 0.6 (-0.2, 1.4) | 0.041 |
|  | Lag2 | -0.1 (-1.0, 0.8) | 0.0 (-0.7, 0.7) | 0.784 |
|  | Lag3 | 0.1 (-0.7, 1.0) | -0.3 (-1.2, 0.5) | 0.407 |
|  | Lag4 | 0.7 (-0.1, 1.4) | -0.7 (-1.5, 0.1) | 0.009 |
|  | 5dMA | -0.1 (-1.5, 1.3) | -0.3 (-1.5, 0.9) | 0.833 |
| ET-1 | Lag0 | -21.0 (-43.8, 1.8) | 5.7 (-13.6, 25.1) | 0.052 |
|  | Lag1 | -14.0 (-37.0, 8.9) | -1.8 (-24.8, 21.2) | 0.412 |
|  | Lag2 | -10.6 (-35.4, 14.1) | -10.3 (-32.8, 12.3) | 0.981 |
|  | Lag3 | -16.2 (-40.7, 8.2) | -25.6 (-50.0, -1.2) | 0.535 |
|  | Lag4 | -19.5 (-41.5, 2.5) | -22.9 (-45.8, 0.0) | 0.805 |
|  | 5dMA | -54.2 (-93.2, -15.1) | -43.1 (-79.8, -6.3) | 0.580 |

Note: 5dMA: 5-day moving average, BADb: brachial artery diameter at baseline, BADhyp: brachial artery diameter at maximum dilatation, CI: confidence interval, ET-1: endothelin-1, FMD: Flow – mediated dilatation, IQR: interquartile range.

**Additional Table 7**. Percent change (95% CI) in HRV markers per IQR increase in NO_2_ in the low and high omega-3 groups.

| Outcome | Lag (Day) | Low omega-3 group | High omega-3 group | *p*_interaction_ |
| --- | --- | --- | --- | --- |
| SDNN | Lag0 | -1.8 (-6.3, 2.9) | 0.7 (-3.1, 4.5) | 0.391 |
|  | Lag1 | -0.3 (-4.6, 4.3) | -0.8 (-5.2, 3.9) | 0.866 |
|  | Lag2 | 1.1 (-3.8, 6.2) | -1.4 (-5.3, 2.7) | 0.389 |
|  | Lag3 | 0.3 (-4.3, 5.1) | 0.6 (-4.0, 5.3) | 0.928 |
|  | Lag4 | 1.1 (-3.0, 5.3) | 0.3 (-3.9, 4.8) | 0.780 |
|  | 5dMA | 0.6 (-6.6, 8.5) | 0.4 (-6.1, 7.4) | 0.959 |
| RMSSD | Lag0 | -2.6 (-9.1, 4.4) | 0.0 (-5.5, 5.9) | 0.539 |
|  | Lag1 | 1.4 (-5.1, 8.3) | 1.6 (-5.1, 8.8) | 0.967 |
|  | Lag2 | 3.3 (-4.0, 11.3) | 1.7 (-4.3, 8.1) | 0.715 |
|  | Lag3 | -0.2 (-7.0, 7.0) | 3.6 (-3.4, 11.0) | 0.398 |
|  | Lag4 | -0.5 (-6.3, 5.6) | 3.1 (-3.2, 9.9) | 0.371 |
|  | 5dMA | 1.3 (-9.5, 13.4) | 5.5 (-4.6, 16.7) | 0.513 |
| HFn | Lag0 | -2.1 (-8.6, 4.4) | -1.9 (-7.2, 3.4) | 0.960 |
|  | Lag1 | -2.9 (-9.0, 3.3) | -4.7 (-11.1, 1.7) | 0.659 |
|  | Lag2 | -2.4 (-9.2, 4.5) | -1.9 (-7.6, 3.8) | 0.904 |
|  | Lag3 | -1.8 (-8.3, 4.6) | -7.2 (-13.6, -0.8) | 0.185 |
|  | Lag4 | -3.9 (-9.7, 1.8) | -1.6 (-7.7, 4.5) | 0.549 |
|  | 5dMA | -8.1 (-18.4, 2.3) | -8.4 (-17.8, 0.9) | 0.946 |
| LFn | Lag0 | 2.4 (-2.8, 7.6) | 2.6 (-1.6, 6.9) | 0.935 |
|  | Lag1 | 3.2 (-1.9, 8.2) | 1.3 (-3.8, 6.5) | 0.585 |
|  | Lag2 | 4.1 (-1.4, 9.6) | -0.8 (-5.4, 3.8) | 0.137 |
|  | Lag3 | 1.2 (-4.1, 6.5) | 3.3 (-1.9, 8.5) | 0.530 |
|  | Lag4 | 2.0 (-2.7, 6.6) | -1.8 (-6.7, 3.1) | 0.221 |
|  | 5dMA | 7.5 (-1.0, 15.9) | 3.2 (-4.4, 10.9) | 0.364 |
| LF/HF | Lag0 | 4.3 (-7.9, 18.2) | 4.9 (-5.3, 16.2) | 0.942 |
|  | Lag1 | 7.2 (-4.8, 20.8) | 7.4 (-5.0, 21.5) | 0.983 |
|  | Lag2 | 7.8 (-5.5, 23.0) | 1.5 (-9.1, 13.3) | 0.438 |
|  | Lag3 | 4.4 (-7.8, 18.3) | 13.4 (0.2, 28.3) | 0.298 |
|  | Lag4 | 7.5 (-3.7, 20.1) | -0.3 (-11.3, 12.1) | 0.305 |
|  | 5dMA | 20.1 (-1.7, 46.7) | 14.7 (-4.2, 37.4) | 0.678 |
| VLF | Lag0 | -21.5 (-34.8, -5.6) | -7.5 (-20.7, 7.7) | 0.151 |
|  | Lag1 | -14.1 (-28.2, 2.7) | 0.8 (-16.2, 21.3) | 0.181 |
|  | Lag2 | -10.7 (-26.7, 8.6) | -7.0 (-21.8, 10.7) | 0.726 |
|  | Lag3 | 13.9 (-5.6, 37.5) | 19.4 (-1.1, 44.2) | 0.691 |
|  | Lag4 | 17.2 (-1.3, 39.2) | 10.9 (-7.6, 33.2) | 0.621 |
|  | 5dMA | -8.4 (-32.5, 24.3) | 4.4 (-21.7, 39.1) | 0.437 |

Note: CI: confidence interval, HFn: normalized high frequency, IQR: interquartile range, LFn: normalized low frequency, LF/HF: low to high frequency ratio, RMSSD: root mean square of successive differences, SDNN: standard deviation standard deviation of normal-to-normal, VLF: very-low frequency.

**Additional Table 8**. Percent change (95% CI) in biomarkers per IQR increase in NO_2_ in the low and high omega-3 groups in two-pollutant models.

| Outcome | Lag (Day) | Adjust for PM_2.5_ | | |  | Adjust for O_3_ | | |
| --- | --- | --- | --- | --- | --- | --- | --- | --- |
|  |  | Low omega-3 group | High omega-3 group | *p*_interaction_ |  | Low omega-3 group | High omega-3 group | *p*_interaction_ |
| FEV1 | Lag0 | 0.5 (-1.0, 2.1) | 0.9 (-0.4, 2.2) | 0.708 |  | 0.6 (-0.9, 2.1) | 0.9 (-0.4, 2.2) | 0.740 |
|  | Lag1 | -0.5 (-2.1, 1.1) | 0.8 (-0.8, 2.4) | 0.184 |  | -0.4 (-1.8, 1.1) | 0.8 (-0.8, 2.3) | 0.240 |
|  | Lag2 | 0.5 (-1.2, 2.1) | 1.4 (0.0, 2.8) | 0.325 |  | 0.3 (-1.3, 1.9) | 1.2 (-0.1, 2.5) | 0.344 |
|  | Lag3 | 0.6 (-0.9, 2.1) | 1.2 (-0.3, 2.6) | 0.536 |  | 0.5 (-1.0, 1.9) | 1.0 (-0.5, 2.4) | 0.581 |
|  | Lag4 | 0.8 (-0.5, 2.1) | 0.3 (-1.2, 1.8) | 0.571 |  | 0.6 (-0.7, 1.9) | 0.1 (-1.3, 1.5) | 0.555 |
|  | 5dMA | 1.6 (-1.0, 4.2) | 2.9 (0.7, 5.1) | 0.353 |  | 1.3 (-1.2, 3.8) | 2.6 (0.5, 4.8) | 0.329 |
| FVC | Lag0 | 0.4 (-0.7, 1.4) | 0.6 (-0.3, 1.5) | 0.693 |  | 0.6 (-0.5, 1.7) | 0.7 (-0.3, 1.6) | 0.923 |
|  | Lag1 | 0.0 (-1.1, 1.1) | 1.0 (-0.2, 2.1) | 0.158 |  | 0.2 (-0.8, 1.3) | 1.1 (0.1, 2.2) | 0.194 |
|  | Lag2 | 1.1 (-0.1, 2.2) | 1.2 (0.2, 2.2) | 0.914 |  | 0.8 (-0.3, 1.9) | 1.0 (0.0, 1.9) | 0.797 |
|  | Lag3 | -0.2 (-1.3, 0.8) | 0.8 (-0.3, 1.9) | 0.118 |  | -0.5 (-1.6, 0.6) | 0.5 (-0.6, 1.6) | 0.138 |
|  | Lag4 | 0.1 (-0.9, 1.0) | 0.8 (-0.3, 1.9) | 0.216 |  | -0.1 (-1.1, 0.8) | 0.6 (-0.4, 1.6) | 0.231 |
|  | 5dMA | 1.0 (-0.9, 3.0) | 2.2 (0.5, 3.9) | 0.239 |  | 0.8 (-1.0, 2.6) | 2.1 (0.4, 3.7) | 0.207 |
| FEV1/FVC | Lag0 | -0.3 (-0.9, 0.4) | -0.2 (-0.7, 0.4) | 0.837 |  | -0.2 (-0.9, 0.4) | -0.1 (-0.6, 0.5) | 0.635 |
|  | Lag1 | -0.6 (-1.2, 0.1) | -0.1 (-0.8, 0.5) | 0.258 |  | -0.5 (-1.1, 0.1) | -0.1 (-0.7, 0.6) | 0.291 |
|  | Lag2 | -0.6 (-1.3, 0.1) | 0.0 (-0.6, 0.5) | 0.151 |  | -0.4 (-1.0, 0.3) | 0.1 (-0.5, 0.6) | 0.261 |
|  | Lag3 | 0.2 (-0.5, 0.9) | 0.0 (-0.6, 0.6) | 0.598 |  | 0.3 (-0.3, 1) | 0.1 (-0.5, 0.7) | 0.533 |
|  | Lag4 | 0.1 (-0.4, 0.7) | 0.1 (-0.5, 0.7) | 0.931 |  | 0.1 (-0.4, 0.7) | 0.1 (-0.5, 0.7) | 0.952 |
|  | 5dMA | -0.5 (-1.6, 0.7) | 0.0 (-1.0, 0.9) | 0.445 |  | -0.2 (-1.3, 0.8) | 0.1 (-0.8, 1.0) | 0.593 |
| Total cholesterol | Lag0 | 0.7 (-1.3, 2.9) | -0.2 (-1.9, 1.5) | 0.454 |  | 0.8 (-1.3, 2.8) | -0.5 (-2.3, 1.2) | 0.308 |
|  | Lag1 | 2.2 (0.1, 4.3) | 0.6 (-1.5, 2.8) | 0.230 |  | 1.2 (-0.7, 3.1) | -0.2 (-2.2, 1.9) | 0.297 |
|  | Lag2 | -0.5 (-2.5, 1.7) | -2.1 (-3.9, -0.3) | 0.174 |  | -0.7 (-2.7, 1.3) | -2.5 (-4.2, -0.7) | 0.149 |
|  | Lag3 | -1.4 (-3.4, 0.7) | 0.3 (-1.8, 2.4) | 0.178 |  | -1.5 (-3.4, 0.5) | 0.1 (-1.9, 2.1) | 0.205 |
|  | Lag4 | 0.2 (-1.6, 2.1) | -0.3 (-2.3, 1.8) | 0.678 |  | -0.2 (-1.9, 1.7) | -0.7 (-2.7, 1.2) | 0.631 |
|  | 5dMA | 0.7 (-2.8, 4.2) | -0.9 (-3.9, 2.3) | 0.408 |  | -0.2 (-3.4, 3.2) | -1.7 (-4.6, 1.3) | 0.413 |
| HDL | Lag0 | 0.8 (-1.4, 3.1) | -0.9 (-2.8, 0.9) | 0.202 |  | 0.6 (-1.6, 2.9) | -1.5 (-3.4, 0.4) | 0.134 |
|  | Lag1 | 0.6 (-1.6, 2.9) | -1.3 (-3.5, 1.0) | 0.152 |  | -0.4 (-2.4, 1.7) | -1.9 (-4.0, 0.2) | 0.252 |
|  | Lag2 | -1.8 (-4.1, 0.5) | -1.6 (-3.6, 0.3) | 0.882 |  | -1.9 (-4.1, 0.4) | -1.7 (-3.6, 0.2) | 0.894 |
|  | Lag3 | -1.4 (-3.6, 0.9) | 0.8 (-1.5, 3.0) | 0.117 |  | -1.6 (-3.7, 0.7) | 0.6 (-1.6, 2.8) | 0.125 |
|  | Lag4 | 0.3 (-1.7, 2.3) | -0.4 (-2.6, 1.9) | 0.620 |  | -0.1 (-2.1, 1.9) | -0.9 (-3.0, 1.2) | 0.559 |
|  | 5dMA | -1.2 (-4.8, 2.6) | -2.3 (-5.4, 1.0) | 0.570 |  | -2.1 (-5.6, 1.5) | -3.0 (-6.0, 0.1) | 0.634 |
| LDL | Lag0 | 0.9 (-2.1, 3.9) | -0.8 (-3.2, 1.7) | 0.364 |  | 1.2 (-1.7, 4.2) | -0.7 (-3.1, 1.8) | 0.298 |
|  | Lag1 | 3.5 (0.5, 6.6) | 1.1 (-1.9, 4.2) | 0.186 |  | 2.1 (-0.6, 4.9) | 0.1 (-2.7, 3.0) | 0.282 |
|  | Lag2 | 0.1 (-2.9, 3.3) | -2.6 (-5.1, -0.1) | 0.115 |  | -0.1 (-3.0, 3.0) | -2.9 (-5.3, -0.4) | 0.102 |
|  | Lag3 | -1.8 (-4.7, 1.1) | 1.0 (-1.9, 4.1) | 0.110 |  | -1.6 (-4.4, 1.3) | 1.2 (-1.6, 4.0) | 0.130 |
|  | Lag4 | -0.5 (-3.1, 2.1) | -0.8 (-3.7, 2.1) | 0.853 |  | -0.7 (-3.1, 1.9) | -0.9 (-3.6, 1.8) | 0.866 |
|  | 5dMA | 0.9 (-4.0, 6.1) | -0.8 (-4.9, 3.6) | 0.524 |  | 0.5 (-4.2, 5.3) | -1.4 (-5.4, 2.8) | 0.476 |

Additional Table 8 continued

| tPA | Lag0 | 2.2 (-3.2, 7.8) | 2.7 (-1.8, 7.4) | 0.874 |  | 1.5 (-3.7, 6.9) | 3.9 (-0.7, 8.6) | 0.475 |
| --- | --- | --- | --- | --- | --- | --- | --- | --- |
|  | Lag1 | 1.1 (-4.2, 6.6) | 0.3 (-5.1, 6.0) | 0.815 |  | 0.5 (-4.3, 5.5) | 0.0 (-5.1, 5.3) | 0.879 |
|  | Lag2 | 1.5 (-4.0, 7.3) | -1.2 (-5.7, 3.5) | 0.390 |  | 1.9 (-3.4, 7.6) | -1.3 (-5.7, 3.2) | 0.312 |
|  | Lag3 | 3.3 (-2.0, 8.9) | -4.0 (-8.9, 1.1) | 0.021 |  | 4.0 (-1.2, 9.5) | -3.1 (-7.9, 1.9) | 0.029 |
|  | Lag4 | 1.3 (-3.3, 6.3) | -0.5 (-5.6, 4.8) | 0.545 |  | 1.3 (-3.2, 6.1) | -0.6 (-5.4, 4.4) | 0.521 |
|  | 5dMA | 5.6 (-3.4, 15.4) | -0.3 (-7.6, 7.7) | 0.220 |  | 5.2 (-3.3, 14.5) | -0.2 (-7.3, 7.4) | 0.257 |
| vWF | Lag0 | 4.4 (-0.4, 9.5) | 1.7 (-2.3, 5.7) | 0.345 |  | 5.6 (0.8, 10.6) | 0.9 (-3.1, 5.0) | 0.124 |
|  | Lag1 | 5.3 (0.4, 10.4) | 7.7 (2.6, 13.2) | 0.424 |  | 4.5 (0.1, 9.1) | 7.1 (2.3, 12.2) | 0.386 |
|  | Lag2 | 2.4 (-2.6, 7.7) | 4.5 (0.2, 9.0) | 0.468 |  | 0.9 (-3.9, 5.9) | 3.1 (-1.0, 7.3) | 0.461 |
|  | Lag3 | -0.4 (-5.1, 4.5) | 6.2 (1.4, 11.4) | 0.026 |  | -1.6 (-6.1, 3.1) | 4.8 (0.1, 9.7) | 0.030 |
|  | Lag4 | -0.3 (-4.4, 4.1) | 0.2 (-4.4, 5.1) | 0.858 |  | -1.0 (-5.0, 3.1) | -0.9 (-5.2, 3.6) | 0.961 |
|  | 5dMA | 8.2 (-0.1, 17.1) | 10 (2.7, 17.9) | 0.679 |  | 5.7 (-2.1, 14.0) | 8.0 (1.0, 15.5) | 0.599 |
| D-dimer | Lag0 | 10.9 (-0.4, 23.5) | 6.0 (-3.2, 16.0) | 0.480 |  | 10.2 (-0.8, 22.4) | 3.5 (-5.5, 13.4) | 0.341 |
|  | Lag1 | 8.7 (-3.6, 22.6) | 2.4 (-9.4, 15.7) | 0.409 |  | 9.6 (-1.7, 22.3) | 2.7 (-8.5, 15.2) | 0.370 |
|  | Lag2 | 0.6 (-11.3, 14.0) | -0.8 (-10.9, 10.4) | 0.844 |  | -1.1 (-12.4, 11.6) | -1.0 (-10.8, 9.8) | 0.991 |
|  | Lag3 | 4.7 (-7.0, 17.9) | -2.6 (-13.5, 9.6) | 0.311 |  | 1.7 (-9.6, 14.3) | -5.2 (-15.5, 6.4) | 0.338 |
|  | Lag4 | 3.7 (-6.6, 15.1) | -1.0 (-11.7, 11.1) | 0.496 |  | 0.2 (-9.5, 11.0) | -5.6 (-15.4, 5.3) | 0.384 |
|  | 5dMA | 19.3 (-1.9, 45.1) | 5.1 (-11.5, 24.9) | 0.212 |  | 11.2 (-7.8, 34.1) | 1.3 (-14.2, 19.7) | 0.362 |
| FMD | Lag0 | -2.2 (-8.0, 3.6) | 0.8 (-4.1, 5.7) | 0.389 |  | -1.3 (-7.0, 4.4) | 0.2 (-4.7, 5.1) | 0.683 |
|  | Lag1 | -1.0 (-6.9, 4.9) | 5.0 (-1.0, 11.1) | 0.093 |  | 0.0 (-5.4, 5.3) | 6.0 (0.4, 11.7) | 0.091 |
|  | Lag2 | -0.7 (-6.9, 5.5) | 6.1 (0.9, 11.4) | 0.053 |  | -1.7 (-7.6, 4.2) | 5.6 (0.6, 10.6) | 0.037 |
|  | Lag3 | -1.9 (-8.0, 4.1) | 2.5 (-3.5, 8.4) | 0.227 |  | -3.0 (-8.9, 2.8) | 1.7 (-4.1, 7.4) | 0.197 |
|  | Lag4 | 2.5 (-2.8, 7.8) | 3.9 (-1.9, 9.6) | 0.696 |  | 2.1 (-3.0, 7.3) | 3.4 (-2.1, 8.8) | 0.718 |
|  | 5dMA | 0.6 (-9.4, 10.6) | 9.3 (0.7, 17.9) | 0.091 |  | -0.2 (-9.7, 9.4) | 8.9 (0.6, 17.2) | 0.079 |
| BADb | Lag0 | -0.2 (-1.1, 0.6) | -0.3 (-1.0, 0.4) | 0.914 |  | -0.1 (-1.0, 0.7) | 0 (-0.8, 0.7) | 0.896 |
|  | Lag1 | -0.5 (-1.4, 0.4) | 0.1 (-0.8, 1.1) | 0.234 |  | -0.4 (-1.2, 0.4) | 0.3 (-0.6, 1.1) | 0.202 |
|  | Lag2 | -0.1 (-1.0, 0.8) | -0.5 (-1.2, 0.3) | 0.462 |  | 0.0 (-0.9, 0.9) | -0.3 (-1.1, 0.4) | 0.578 |
|  | Lag3 | 0.1 (-0.8, 1.0) | -0.6 (-1.4, 0.3) | 0.202 |  | 0.4 (-0.5, 1.2) | -0.3 (-1.1, 0.5) | 0.207 |
|  | Lag4 | 0.4 (-0.4, 1.2) | -1.0 (-1.9, -0.2) | 0.004 |  | 0.6 (-0.2, 1.3) | -0.8 (-1.6, 0.0) | 0.007 |
|  | 5dMA | -0.4 (-1.9, 1.1) | -1.0 (-2.3, 0.3) | 0.468 |  | 0.0 (-1.5, 1.4) | -0.7 (-1.9, 0.5) | 0.398 |
| BADhyp | Lag0 | -0.4 (-1.3, 0.5) | -0.3 (-1.1, 0.4) | 0.932 |  | -0.2 (-1.0, 0.6) | -0.1 (-0.8, 0.6) | 0.866 |
|  | Lag1 | -0.6 (-1.5, 0.2) | 0.5 (-0.4, 1.4) | 0.040 |  | -0.5 (-1.2, 0.3) | 0.7 (-0.2, 1.5) | 0.032 |
|  | Lag2 | -0.2 (-1.1, 0.7) | -0.1 (-0.8, 0.7) | 0.820 |  | -0.2 (-1.1, 0.7) | 0.0 (-0.7, 0.8) | 0.646 |
|  | Lag3 | -0.1 (-1.0, 0.8) | -0.5 (-1.4, 0.3) | 0.400 |  | 0.1 (-0.7, 1.0) | -0.3 (-1.2, 0.5) | 0.420 |
|  | Lag4 | 0.5 (-0.3, 1.3) | -0.9 (-1.7, 0.0) | 0.007 |  | 0.7 (-0.1, 1.4) | -0.7 (-1.5, 0.1) | 0.009 |
|  | 5dMA | -0.6 (-2.1, 0.8) | -0.6 (-1.9, 0.6) | 0.978 |  | -0.2 (-1.7, 1.2) | -0.3 (-1.5, 0.9) | 0.912 |
| ET-1 | Lag0 | -21.5 (-45.4, 2.4) | 6.3 (-13.8, 26.4) | 0.043 |  | -20.1 (-43.7, 3.4) | 6.8 (-13.5, 27.1) | 0.055 |
|  | Lag1 | -19.9 (-45.3, 5.5) | -7.1 (-32.2, 17.9) | 0.394 |  | -14.3 (-37.4, 8.7) | 0.1 (-23.2, 23.4) | 0.333 |
|  | Lag2 | -6.0 (-32.4, 20.4) | -6.8 (-30.7, 17.0) | 0.956 |  | -11.3 (-36.2, 13.6) | -6.6 (-29.4, 16.3) | 0.747 |
|  | Lag3 | -15.1 (-40.7, 10.5) | -25.0 (-50.7, 0.8) | 0.515 |  | -15.9 (-40.6, 8.8) | -24.6 (-49.3, 0.0) ^*^ | 0.565 |
|  | Lag4 | -17.6 (-40.4, 5.3) | -20.2 (-44.6, 4.1) | 0.849 |  | -19.2 (-41.2, 2.8) | -21.8 (-44.8, 1.3) | 0.853 |
|  | 5dMA | -53.1 (-94.9, -11.3) | -42.2 (-80.7, -3.7) | 0.591 |  | -54.8 (-94.4, -15.2) | -43.2 (-80.1, -6.4) | 0.567 |

Additional Table 8 continued

| SDNN | Lag0 | -1.3 (-6.0, 3.6) | 1.1 (-2.8, 5.3) | 0.403 |  | -2.4 (-6.9, 2.3) | -0.2 (-4.1, 3.9) | 0.446 |
| --- | --- | --- | --- | --- | --- | --- | --- | --- |
|  | Lag1 | 0.4 (-4.4, 5.4) | -0.1 (-5.0, 5.0) | 0.865 |  | -0.3 (-4.7, 4.2) | -0.5 (-5.1, 4.3) | 0.951 |
|  | Lag2 | 0.2 (-4.9, 5.5) | -1.9 (-6, 2.4) | 0.482 |  | 1.2 (-3.8, 6.4) | -1.0 (-5.0, 3.2) | 0.463 |
|  | Lag3 | -0.3 (-5.0, 4.7) | 0.0 (-4.7, 4.9) | 0.926 |  | 0.0 (-4.6, 4.8) | 0.4 (-4.1, 5.2) | 0.873 |
|  | Lag4 | 0.8 (-3.4, 5.2) | -0.1 (-4.7, 4.7) | 0.747 |  | 1.1 (-3.0, 5.3) | 0.0 (-4.3, 4.4) | 0.690 |
|  | 5dMA | 0.3 (-7.4, 8.7) | 0.2 (-6.6, 7.5) | 0.978 |  | 0.1 (-7.3, 8.0) | 0.3 (-6.2, 7.3) | 0.950 |
| RMSSD | Lag0 | -1.9 (-8.8, 5.5) | 0.9 (-5.0, 7.2) | 0.512 |  | -3 (-9.6, 4.1) | -1.2 (-6.9, 5.0) | 0.668 |
|  | Lag1 | 2.3 (-4.9, 10.0) | 2.5 (-4.9, 10.5) | 0.964 |  | 1.1 (-5.5, 8.0) | 1.0 (-5.9, 8.4) | 0.989 |
|  | Lag2 | 2.3 (-5.3, 10.6) | 1.5 (-4.8, 8.3) | 0.855 |  | 3.2 (-4.3, 11.3) | 1.8 (-4.4, 8.4) | 0.766 |
|  | Lag3 | -0.5 (-7.5, 7.1) | 3.3 (-3.9, 11.1) | 0.401 |  | -0.8 (-7.6, 6.6) | 3.3 (-3.6, 10.8) | 0.362 |
|  | Lag4 | -0.3 (-6.4, 6.1) | 3.4 (-3.4, 10.7) | 0.365 |  | -0.5 (-6.3, 5.6) | 2.8 (-3.6, 9.5) | 0.418 |
|  | 5dMA | 1.7 (-9.9, 14.7) | 5.8 (-4.8, 17.5) | 0.530 |  | 0.1 (-10.7, 12.2) | 5.3 (-4.7, 16.5) | 0.419 |
| HFn | Lag0 | -1.0 (-7.7, 5.8) | -0.3 (-5.9, 5.2) | 0.878 |  | -1.9 (-8.5, 4.8) | -2.5 (-8.2, 3.1) | 0.875 |
|  | Lag1 | -2.8 (-9.5, 4.0) | -4.5 (-11.5, 2.4) | 0.670 |  | -3.1 (-9.3, 3.1) | -4.7 (-11.3, 1.8) | 0.693 |
|  | Lag2 | -2.0 (-9.2, 5.2) | -1.2 (-7.3, 4.8) | 0.854 |  | -2.2 (-9.1, 4.8) | -1.6 (-7.4, 4.2) | 0.890 |
|  | Lag3 | -2.0 (-8.8, 4.7) | -7.4 (-14.1, -0.7) | 0.187 |  | -2.5 (-9.1, 4.0) | -7.5 (-13.9, -1.1) | 0.220 |
|  | Lag4 | -4.6 (-10.6, 1.4) | -2.5 (-9.0, 4.0) | 0.595 |  | -3.9 (-9.7, 1.8) | -1.9 (-8.0, 4.2) | 0.592 |
|  | 5dMA | -7.4 (-18.5, 3.7) | -8 (-17.7, 1.7) | 0.919 |  | -8.8 (-19.3, 1.8) | -8.6 (-17.9, 0.8) | 0.973 |
| LFn | Lag0 | 1.3 (-4.1, 6.7) | 1.3 (-3.2, 5.8) | 0.988 |  | 2.4 (-3.0, 7.8) | 2.5 (-2.1, 7.1) | 0.967 |
|  | Lag1 | 2.7 (-2.8, 8.2) | 0.8 (-4.8, 6.5) | 0.577 |  | 3.5 (-1.5, 8.5) | 2.5 (-2.8, 7.7) | 0.765 |
|  | Lag2 | 3.6 (-2.2, 9.4) | -1.5 (-6.5, 3.4) | 0.119 |  | 4.4 (-1.2, 10.0) | -0.1 (-4.8, 4.6) | 0.171 |
|  | Lag3 | 1.7 (-3.9, 7.2) | 3.7 (-1.7, 9.2) | 0.533 |  | 1.6 (-3.8, 6.9) | 3.5 (-1.8, 8.7) | 0.568 |
|  | Lag4 | 2.5 (-2.3, 7.4) | -1.1 (-6.3, 4.2) | 0.248 |  | 2.0 (-2.6, 6.6) | -1.5 (-6.4, 3.4) | 0.255 |
|  | 5dMA | 6.9 (-2.1, 16.0) | 2.9 (-5.1, 10.8) | 0.387 |  | 8.4 (-0.1, 17.0) | 3.4 (-4.2, 11.0) | 0.286 |
| LF/HF | Lag0 | 1.4 (-10.9, 15.4) | 1.4 (-9.0, 12.9) | 0.999 |  | 4.0 (-8.6, 18.2) | 5.8 (-5.2, 18.0) | 0.827 |
|  | Lag1 | 6.3 (-6.7, 21.2) | 6.4 (-7.0, 21.7) | 0.992 |  | 7.8 (-4.4, 21.6) | 8.4 (-4.5, 23.0) | 0.950 |
|  | Lag2 | 6.6 (-7.3, 22.5) | -0.3 (-11.3, 12.1) | 0.398 |  | 7.9 (-5.7, 23.4) | 1.6 (-9.2, 13.7) | 0.449 |
|  | Lag3 | 5.1 (-7.8, 19.8) | 14.1 (0.2, 29.9) | 0.300 |  | 5.7 (-6.9, 19.9) | 14.0 (0.7, 29.0) | 0.339 |
|  | Lag4 | 8.8 (-3.0, 22.0) | 1.3 (-10.6, 14.8) | 0.336 |  | 7.6 (-3.6, 20.1) | 0.3 (-10.8, 12.8) | 0.343 |
|  | 5dMA | 17.9 (-4.9, 46.1) | 13.2 (-6.2, 36.7) | 0.719 |  | 22.3 (-0.2, 49.8) | 15.1 (-3.9, 37.8) | 0.585 |
| VLF | Lag0 | -17.6 (-31.9, -0.2) | -4.3 (-18.4, 12.3) | 0.187 |  | -19.7 (-33.6, -3.0) | -5.4 (-19.5, 11.2) | 0.159 |
|  | Lag1 | -11.2 (-27.1, 8.1) | 3.7 (-15.3, 26.9) | 0.195 |  | -14.8 (-28.8, 2.0) | -1.9 (-18.7, 18.3) | 0.238 |
|  | Lag2 | -7.3 (-24.8, 14.4) | -2.0 (-18.8, 18.3) | 0.643 |  | -11.6 (-27.7, 8.0) | -6.4 (-21.7, 11.9) | 0.627 |
|  | Lag3 | 9.5 (-10.1, 33.3) | 15.4 (-5.3, 40.7) | 0.657 |  | 11.2 (-8, 34.3) | 18.7 (-1.7, 43.2) | 0.580 |
|  | Lag4 | 18.1 (-1.2, 41.2) | 13.4 (-6.7, 38.0) | 0.720 |  | 16.0 (-2.3, 37.7) | 11.0 (-7.7, 33.5) | 0.697 |
|  | 5dMA | -2.8 (-29.9, 34.8) | 10.3 (-18.3, 48.9) | 0.454 |  | -7.6 (-32.3, 26) | 5.8 (-20.7, 41.1) | 0.422 |

Note: the effect estimates of biomarkers per IQR increase in NO_2_ were adjusted by PM_2.5_ or O_3_. BADb: brachial artery diameter at baseline, BADhyp: brachial artery diameter at maximum dilatation, CI: confidence interval, ET-1: endothelin-1, FEV1: forced expiratory volume at the end of the first second, FMD: Flow – mediated dilatation, FVC: forced vital capacity, HDL: high-density lipoproteins, HFn: normalized high frequency, IQR: interquartile range, LDL: low-density lipoproteins, LFn: normalized low frequency, LF/HF: low to high frequency ratio, RMSSD: root mean square of successive differences, SDNN: standard deviation standard deviation of normal-to-normal, tPA: tissue plasminogen activator, VLF: very-low frequency, vWF: von Willebrand factor.

**Additional Table 9**. Percent change (95% CI) in biomarkers per IQR increase in NO_2_ in the low and high omega-3 groups after excluding outliers.

| Outcome | Lag (Day) | Low omega-3 group | High omega-3 group | *p*_interaction_ |
| --- | --- | --- | --- | --- |
| tPA | Lag0 | -0.5 (-5.7, 4.9) | 3.0 (-1.2, 7.4) | 0.279 |
|  | Lag1 | -2.1 (-7.1, 3.3) | -0.5 (-5.3, 4.7) | 0.635 |
|  | Lag2 | 1.2 (-4.6, 7.3) | -1.2 (-5.6, 3.5) | 0.505 |
|  | Lag3 | 4.1 (-1.9, 10.4) | -2.4 (-7.1, 2.5) | 0.070 |
|  | Lag4 | -0.5 (-5.3, 4.6) | -2.3 (-7.3, 2.8) | 0.573 |
|  | 5dMA | 0.8 (-8.8, 11.4) | -1.0 (-8.1, 6.6) | 0.724 |
| D-dimer | Lag0 | 4.1 (-3.5, 12.3) | 2.9 (-3.4, 9.5) | 0.797 |
|  | Lag1 | -0.1 (-7.3, 7.8) | 1.3 (-6.4, 9.6) | 0.793 |
|  | Lag2 | 8.4 (0.1, 17.4) | 1.9 (-4.8, 9.1) | 0.188 |
|  | Lag3 | 8.0 (-0.1, 16.7) | 1.2 (-6.3, 9.2) | 0.179 |
|  | Lag4 | 0.9 (-5.8, 8.1) | -0.1 (-7.2, 7.5) | 0.832 |
|  | 5dMA | 12.0 (-1.1, 27.0) | 5.1 (-6.2, 17.7) | 0.352 |
| SDNN | Lag0 | -1.7 (-6.1, 3.0) | 0.7 (-3.0, 4.6) | 0.400 |
|  | Lag1 | -0.1 (-4.4, 4.4) | -0.4 (-4.9, 4.3) | 0.920 |
|  | Lag2 | 1.3 (-3.6, 6.3) | -1.0 (-5.0, 3.1) | 0.429 |
|  | Lag3 | 0.2 (-4.4, 5.0) | 0.9 (-3.7, 5.7) | 0.816 |
|  | Lag4 | 1.1 (-3.0, 5.3) | 0.6 (-3.6, 5.1) | 0.877 |
|  | 5dMA | 0.9 (-6.4, 8.8) | 1.2 (-5.5, 8.2) | 0.950 |
| RMSSD | Lag0 | -2.5 (-9.1, 4.5) | 0.4 (-5.2, 6.3) | 0.501 |
|  | Lag1 | 1.4 (-5.1, 8.4) | 2.3 (-4.6, 9.6) | 0.852 |
|  | Lag2 | 3.0 (-4.4, 11.0) | 2.0 (-4.2, 8.6) | 0.826 |
|  | Lag3 | -0.7 (-7.5, 6.6) | 3.8 (-3.3, 11.4) | 0.329 |
|  | Lag4 | -0.6 (-6.4, 5.6) | 3.5 (-2.9, 10.4) | 0.319 |
|  | 5dMA | 1.0 (-9.8, 13.2) | 6.4 (-3.9, 17.8) | 0.411 |
| LF/HF | Lag0 | 4.0 (-7.7, 17.2) | 3.5 (-6.2, 14.2) | 0.953 |
|  | Lag1 | 6.0 (-5.5, 18.9) | 5.0 (-6.8, 18.3) | 0.902 |
|  | Lag2 | 7.1 (-5.7, 21.5) | 0.1 (-10.0, 11.3) | 0.369 |
|  | Lag3 | 2.8 (-8.9, 15.9) | 12.3 (-0.4, 26.5) | 0.244 |
|  | Lag4 | 6.0 (-4.7, 17.9) | 0.5 (-10.2, 12.4) | 0.454 |
|  | 5dMA | 16.2 (-4.3, 41.0) | 11.5 (-6.3, 32.7) | 0.698 |
| VLF | Lag0 | -19.7 (-32.9, -3.9) | -2.3 (-16.0, 13.6) | 0.076 |
|  | Lag1 | -14.8 (-28.3, 1.3) | -0.1 (-16.5, 19.4) | 0.170 |
|  | Lag2 | -8.2 (-24.1, 11.2) | -6.8 (-21.2, 10.2) | 0.900 |
|  | Lag3 | 16.7 (-2.7, 40.0) | 15.1 (-4.2, 38.4) | 0.907 |
|  | Lag4 | 14.4 (-3.1, 35.0) | 5.1 (-12.6, 26.4) | 0.445 |
|  | 5dMA | -6.0 (-30.0, 26.2) | 4.5 (-20.9, 38.0) | 0.516 |

Note: this table only presents outcomes that have outliers excluded. CI: confidence interval, IQR: interquartile range, LF/HF: low to high frequency ratio, RMSSD: root mean square of successive differences, SDNN: standard deviation standard deviation of normal-to-normal, tPA: tissue plasminogen activator, VLF: very-low frequency.

**Additional Table 10**. Percent change (95% CI) in biomarkers per IQR increase in NO_2_ in the low and high omega-3 groups after adding marital status and education as covariates.

| Outcome | Lag (Day) | Low omega-3 group | High omega-3 group | *p*_interaction_ |
| --- | --- | --- | --- | --- |
| FEV1 | Lag0 | 0.9 (-0.6, 2.4) | 1 (-0.2, 2.3) | 0.85 |
|  | Lag1 | -0.3 (-1.8, 1.1) | 0.9 (-0.6, 2.4) | 0.19 |
|  | Lag2 | 0.3 (-1.3, 1.8) | 1.2 (-0.1, 2.5) | 0.32 |
|  | Lag3 | 0.3 (-1.2, 1.8) | 0.9 (-0.6, 2.3) | 0.54 |
|  | Lag4 | 0.5 (-0.7, 1.8) | 0 (-1.4, 1.4) | 0.52 |
|  | 5dMA | 1.2 (-1.3, 3.6) | 2.6 (0.4, 4.7) | 0.30 |
| FVC | Lag0 | 0.7 (-0.4, 1.8) | 0.9 (0, 1.8) | 0.78 |
|  | Lag1 | 0.3 (-0.7, 1.3) | 1.2 (0.2, 2.2) | 0.18 |
|  | Lag2 | 0.8 (-0.2, 1.9) | 1 (0, 1.9) | 0.83 |
|  | Lag3 | -0.6 (-1.7, 0.4) | 0.4 (-0.6, 1.5) | 0.11 |
|  | Lag4 | -0.2 (-1.1, 0.8) | 0.5 (-0.5, 1.5) | 0.26 |
|  | 5dMA | 0.8 (-1, 2.6) | 2.1 (0.5, 3.7) | 0.20 |
| FEV1/FVC | Lag0 | -0.1 (-0.8, 0.5) | -0.1 (-0.6, 0.4) | 0.99 |
|  | Lag1 | -0.5 (-1.1, 0.1) | -0.1 (-0.7, 0.5) | 0.28 |
|  | Lag2 | -0.5 (-1.1, 0.2) | 0 (-0.5, 0.6) | 0.19 |
|  | Lag3 | 0.3 (-0.4, 0.9) | 0.1 (-0.6, 0.7) | 0.58 |
|  | Lag4 | 0.1 (-0.4, 0.7) | 0.1 (-0.5, 0.7) | 0.94 |
|  | 5dMA | -0.3 (-1.3, 0.7) | 0.1 (-0.9, 1) | 0.52 |
| Total Cholesterol | Lag0 | 0.8 (-1.1, 2.9) | 0 (-1.7, 1.6) | 0.48 |
|  | Lag1 | 1 (-0.9, 2.9) | -0.4 (-2.4, 1.6) | 0.28 |
|  | Lag2 | -1.1 (-3.1, 0.9) | -2.6 (-4.3, -0.9) | 0.23 |
|  | Lag3 | -1.7 (-3.7, 0.2) | 0 (-2, 2) | 0.17 |
|  | Lag4 | -0.2 (-2, 1.6) | -0.9 (-2.8, 1.1) | 0.58 |
|  | 5dMA | -0.8 (-3.9, 2.5) | -1.7 (-4.7, 1.3) | 0.59 |
| HDL | Lag0 | 0.5 (-1.7, 2.7) | -1.2 (-3, 0.6) | 0.21 |
|  | Lag1 | -0.6 (-2.6, 1.4) | -2.4 (-4.5, -0.3) | 0.18 |
|  | Lag2 | -2.3 (-4.4, -0.1) | -1.9 (-3.7, -0.1) | 0.80 |
|  | Lag3 | -1.6 (-3.8, 0.5) | 0.6 (-1.5, 2.8) | 0.11 |
|  | Lag4 | -0.1 (-2.1, 1.8) | -0.9 (-2.9, 1.2) | 0.57 |
|  | 5dMA | -2.6 (-6, 0.9) | -3 (-6, 0.1) | 0.82 |
| LDL | Lag0 | 1.1 (-1.7, 4) | -0.5 (-2.8, 1.8) | 0.36 |
|  | Lag1 | 1.8 (-0.9, 4.5) | -0.4 (-3.2, 2.5) | 0.24 |
|  | Lag2 | -0.5 (-3.4, 2.4) | -3.1 (-5.5, -0.7) | 0.14 |
|  | Lag3 | -1.9 (-4.7, 0.9) | 1 (-1.8, 3.9) | 0.11 |
|  | Lag4 | -0.7 (-3.2, 1.9) | -1.1 (-3.7, 1.7) | 0.82 |
|  | 5dMA | -0.3 (-4.9, 4.5) | -1.6 (-5.6, 2.6) | 0.64 |
|  |  |  |  |  |
|  |  |  |  |  |
|  |  |  |  |  |
| Additional Table 10 continued | | | | |
| tPA | Lag0 | 2.2 (-2.9, 7.6) | 2.4 (-1.9, 6.8) | 0.96 |
|  | Lag1 | 0.4 (-4.3, 5.4) | -0.5 (-5.4, 4.8) | 0.79 |
|  | Lag2 | 1.8 (-3.4, 7.4) | -1.7 (-6, 2.7) | 0.26 |
|  | Lag3 | 4.6 (-0.6, 10.1) | -2.9 (-7.7, 2.1) | 0.02 |
|  | Lag4 | 1.4 (-3.1, 6.2) | -0.5 (-5.3, 4.4) | 0.53 |
|  | 5dMA | 5.9 (-2.5, 15.2) | -0.3 (-7.4, 7.4) | 0.19 |
| vWF | Lag0 | 5.2 (0.5, 10.1) | 2.5 (-1.3, 6.4) | 0.36 |
|  | Lag1 | 4.1 (-0.3, 8.7) | 6.8 (2.1, 11.8) | 0.37 |
|  | Lag2 | 0 (-4.7, 4.9) | 2.7 (-1.3, 7) | 0.35 |
|  | Lag3 | -2.1 (-6.6, 2.6) | 4.8 (0.1, 9.7) | 0.02 |
|  | Lag4 | -1 (-5, 3.2) | -0.8 (-5.1, 3.7) | 0.92 |
|  | 5dMA | 4.7 (-2.9, 12.9) | 8.3 (1.3, 15.8) | 0.42 |
| D-dimer | Lag0 | 11 (0.2, 23.1) | 6 (-2.7, 15.5) | 0.47 |
|  | Lag1 | 10.3 (-1.1, 22.9) | 4 (-7.1, 16.4) | 0.42 |
|  | Lag2 | -0.3 (-11.5, 12.2) | -0.6 (-10.2, 10) | 0.97 |
|  | Lag3 | 2.2 (-9, 14.7) | -5.3 (-15.6, 6.3) | 0.30 |
|  | Lag4 | -0.5 (-10.2, 10.2) | -5.8 (-15.5, 5.1) | 0.43 |
|  | 5dMA | 12.8 (-6.3, 35.7) | 1.8 (-13.9, 20.3) | 0.31 |
| FMD | Lag0 | -1.7 (-7.3, 4) | 0.8 (-3.9, 5.5) | 0.47 |
|  | Lag1 | -0.1 (-5.4, 5.2) | 5.9 (0.4, 11.5) | 0.09 |
|  | Lag2 | -0.9 (-6.8, 4.9) | 5.5 (0.6, 10.4) | 0.06 |
|  | Lag3 | -2.5 (-8.3, 3.3) | 1.5 (-4.2, 7.2) | 0.27 |
|  | Lag4 | 2 (-3.2, 7.1) | 3 (-2.4, 8.4) | 0.77 |
|  | 5dMA | 0.4 (-9, 9.7) | 8.6 (0.3, 16.9) | 0.11 |
| BADb | Lag0 | -0.2 (-1, 0.7) | -0.2 (-0.9, 0.5) | 0.91 |
|  | Lag1 | -0.5 (-1.3, 0.3) | 0.2 (-0.7, 1) | 0.23 |
|  | Lag2 | 0 (-0.9, 0.9) | -0.3 (-1.1, 0.4) | 0.54 |
|  | Lag3 | 0.4 (-0.5, 1.2) | -0.3 (-1.1, 0.5) | 0.21 |
|  | Lag4 | 0.6 (-0.2, 1.3) | -0.8 (-1.6, 0) | 0.01 |
|  | 5dMA | 0 (-1.4, 1.4) | -0.7 (-1.9, 0.5) | 0.36 |
| BADhyp | Lag0 | -0.3 (-1.1, 0.5) | -0.3 (-0.9, 0.4) | 0.96 |
|  | Lag1 | -0.5 (-1.3, 0.3) | 0.6 (-0.2, 1.4) | 0.04 |
|  | Lag2 | -0.2 (-1.1, 0.7) | 0 (-0.7, 0.7) | 0.72 |
|  | Lag3 | 0.1 (-0.7, 1) | -0.3 (-1.1, 0.5) | 0.41 |
|  | Lag4 | 0.7 (-0.1, 1.4) | -0.6 (-1.4, 0.2) | 0.01 |
|  | 5dMA | -0.2 (-1.6, 1.3) | -0.3 (-1.5, 0.9) | 0.86 |
| ET-1 | Lag0 | -21.9 (-44.7, 0.9) | 8.9 (-10.5, 28.3) | 0.03 |
|  | Lag1 | -15 (-38, 7.9) | -2.1 (-25.2, 20.9) | 0.39 |
|  | Lag2 | -11.7 (-36.5, 13.1) | -8.6 (-31.2, 13.9) | 0.84 |
|  | Lag3 | -15.6 (-40.1, 8.9) | -25.3 (-49.8, -0.7) | 0.52 |
|  | Lag4 | -20.9 (-43, 1.1) | -21.8 (-44.8, 1.1) | 0.95 |
|  | 5dMA | -54.9 (-94, -15.8) | -39.5 (-76.4, -2.6) | 0.45 |
| Additional Table 10 continued | | | | |
| SDNN | Lag0 | -2 (-6.4, 2.7) | 0.6 (-3.1, 4.5) | 0.37 |
|  | Lag1 | -0.3 (-4.6, 4.3) | -0.8 (-5.3, 3.9) | 0.86 |
|  | Lag2 | 0.9 (-3.9, 6) | -1.4 (-5.3, 2.7) | 0.43 |
|  | Lag3 | 0.3 (-4.3, 5.1) | 0.8 (-3.8, 5.5) | 0.88 |
|  | Lag4 | 1.3 (-2.8, 5.5) | 0.4 (-3.8, 4.9) | 0.76 |
|  | 5dMA | 0.6 (-6.7, 8.5) | 0.5 (-6, 7.6) | 0.98 |
| RMSSD | Lag0 | -2.8 (-9.3, 4.2) | 0 (-5.5, 5.9) | 0.51 |
|  | Lag1 | 1.3 (-5.2, 8.3) | 1.4 (-5.3, 8.6) | 0.98 |
|  | Lag2 | 3 (-4.3, 11) | 1.7 (-4.3, 8.2) | 0.77 |
|  | Lag3 | -0.2 (-7, 7.1) | 3.9 (-3.1, 11.3) | 0.36 |
|  | Lag4 | -0.3 (-6.1, 5.8) | 3.4 (-3, 10.2) | 0.36 |
|  | 5dMA | 1.1 (-9.6, 13.2) | 5.7 (-4.4, 16.9) | 0.48 |
| HFn | Lag0 | -2.3 (-8.7, 4.1) | -2 (-7.3, 3.3) | 0.94 |
|  | Lag1 | -3.1 (-9.2, 3.1) | -5.2 (-11.5, 1.2) | 0.61 |
|  | Lag2 | -2.9 (-9.7, 3.9) | -1.8 (-7.5, 3.8) | 0.80 |
|  | Lag3 | -1.5 (-8, 4.9) | -6.7 (-13.1, -0.3) | 0.20 |
|  | Lag4 | -3.6 (-9.3, 2.1) | -1.2 (-7.3, 4.9) | 0.53 |
|  | 5dMA | -8.1 (-18.4, 2.2) | -8.3 (-17.6, 1) | 0.97 |
| LFn | Lag0 | 2.5 (-2.7, 7.7) | 2.8 (-1.5, 7.1) | 0.93 |
|  | Lag1 | 3.2 (-1.8, 8.2) | 1.7 (-3.4, 6.9) | 0.65 |
|  | Lag2 | 4.6 (-0.9, 10.1) | -0.9 (-5.5, 3.8) | 0.10 |
|  | Lag3 | 0.9 (-4.4, 6.2) | 2.8 (-2.4, 8) | 0.57 |
|  | Lag4 | 1.6 (-3, 6.3) | -2.2 (-7.1, 2.7) | 0.22 |
|  | 5dMA | 7.4 (-1, 15.8) | 3.1 (-4.5, 10.7) | 0.35 |
| LF/HF | Lag0 | 4.7 (-7.6, 18.5) | 5.2 (-5, 16.5) | 0.95 |
|  | Lag1 | 7.5 (-4.6, 21) | 8.4 (-4.2, 22.5) | 0.92 |
|  | Lag2 | 8.9 (-4.5, 24.2) | 1.4 (-9.2, 13.1) | 0.36 |
|  | Lag3 | 3.8 (-8.4, 17.5) | 12.1 (-0.9, 26.9) | 0.32 |
|  | Lag4 | 6.8 (-4.3, 19.3) | -1.1 (-12, 11.2) | 0.30 |
|  | 5dMA | 20.1 (-1.6, 46.6) | 14.4 (-4.5, 37) | 0.66 |
| VLF | Lag0 | -21.3 (-34.6, -5.3) | -7.7 (-20.9, 7.6) | 0.17 |
|  | Lag1 | -13.1 (-27.4, 4) | 0.9 (-16.2, 21.4) | 0.21 |
|  | Lag2 | -9.5 (-25.8, 10.4) | -4.5 (-20, 14.1) | 0.65 |
|  | Lag3 | 12.1 (-7.3, 35.5) | 19.3 (-1.3, 44.2) | 0.60 |
|  | Lag4 | 17 (-1.5, 39) | 10.8 (-7.7, 33.1) | 0.63 |
|  | 5dMA | -7.8 (-32.1, 25.2) | 6.2 (-20.4, 41.7) | 0.40 |

Note: the effect estimates of biomarkers per IQR increase in NO_2_ were adjusted by adding marital status and education level as covariates besides the ones already in the model. BADb: brachial artery diameter at baseline, BADhyp: brachial artery diameter at maximum dilatation, CI: confidence interval, ET-1: endothelin-1, FEV1: forced expiratory volume at the end of the first second, FMD: Flow – mediated dilatation, FVC: forced vital capacity, HDL: high-density lipoproteins, HFn: normalized high frequency, IQR: interquartile range, LDL: low-density lipoproteins, LFn: normalized low frequency, LF/HF: low to high frequency ratio, RMSSD: root mean square of successive differences, SDNN: standard deviation standard deviation of normal-to-normal, tPA: tissue plasminogen activator, VLF: very-low frequency, vWF: von Willebrand factor.
